# Structural Effects on the Antioxidant Properties of Amino Acid Betaxanthins

**DOI:** 10.3390/antiox11112259

**Published:** 2022-11-16

**Authors:** Larissa Cerrato Esteves, Caroline Oliveira Machado, Letícia Christina Pires Gonçalves, Victor Fernandes Cavalcante, Guilherme Obeid, Thiago Carita Correra, Erick Leite Bastos

**Affiliations:** Departamento de Química Fundamental, Instituto de Química, Universidade de São Paulo, São Paulo 05508-000, SP, Brazil

**Keywords:** betalains, betaxanthins, amino acids, semisynthesis, plant pigments

## Abstract

Betaxanthins are natural products with high antioxidant and anti-inflammatory properties. Here, we describe the semisynthesis of twenty-one betaxanthins derived from proteinogenic amino acids, including the elusive betaxanthin of l-cysteine and two betaxanthins derived from l-lysine, and rationalize their antioxidant properties in mechanistic terms. The antioxidant capacity and redox potential of these betaxanthins were compared to those of model betaxanthins derived from dopamine, l-DOPA (L-3,4-dihydroxyphenylalanine), and pyrrolidine and structure–property relationships were established by using matched molecular pair analysis and a model developed using a genetic algorithm. Either a phenol or indole moiety enhance the antioxidant capacity of betaxanthins, which is overall much higher than that of their amino acid precursors and standard antioxidants, except for the cysteine-betaxanthin. The one-electron oxidation of amino acid betaxanthins produces radicals stabilized in multiple centers, as demonstrated by quantum chemical calculations.

## 1. Introduction

The gradual increase in oxygen levels in the atmosphere forced primordial living organisms to fixate amino acids capable of meeting the challenges posed by reactive oxygen species to their cells [1]. Tryptophan (Trp, W), tyrosine (Tyr, Y), methionine (Met, M), and cysteine (Cys, C), for example, are more reactive toward peroxyl radicals than amino acids that appeared earlier, such as phenylalanine (Phe, F), glutamic acid (Glu, E), and proline (Pro, P) [2]. The diversification of the amino acid repertoire gave living organisms the tools to adapt to biotic and abiotic stress, and their colors [3]. Natural pigments biosynthesized from amino acids often have antioxidant properties, finding application as safe additives for foods and cosmetics. Such molecules evolved under harsh environmental conditions and adapted to resist oxidative distress [4], often at the cost of losing any structural resemblance to their precursor amino acids. Betalains are important exceptions in which amino acids are incorporated during biosynthesis without decarboxylation, deamination, or major changes, except for the amino group becoming part of a conjugated imine–enamine system [5].

Betalains are l-tyrosine-derived water-soluble pigments that impart color to most plants of the Caryophyllales order [6,7,8], some fungi of the genera *Amanita*, *Hygrocybe*, and *Hygrophorus* [9,10,11], and the *Gluconacetobacter diazotrophicus* bacterium [12]. Betalain-rich plants show high anti-inflammatory and antiproliferative properties, and have health-promoting effects in humans [13,14]. Betalamic acid is the key biosynthetic precursor of betalains, which were originally classified according to their colors as red–purple betacyanins or yellow betaxanthins [15]. The betaxanthins of all proteinogenic α-amino acids, except l-cysteine, have been found in living organisms [16,17,18,19,20,21,22,23,24,25,26]. Schliemann and coauthors demonstrated that these betaxanthins result from the spontaneous coupling between betalamic acid and amino acids and developed a method for their non-stereoselective semisynthesis in vitro using a scalemic mixture of betalamic acid enriched in the *S*-enantiomer that was extracted from red beetroot [*Beta vulgaris* (L.)] juice submitted to alkaline hydrolysis [27]. Notably, there is still no report on the isolation and characterization of the l-cysteine-betaxanthin, despite the use of this amino acid to preserve betalains in fresh-cut red beetroots [28].

DOPA dioxygenases (DODA) can be used to produce betalamic acid from the oxidation of l-DOPA by molecular oxygen [29,30,31]. Sekiguchi et al. used the recombinant DODA of *Mirabilis jalapa* (L.) to synthesize amino acid betaxanthins and found that indicaxanthin (Pro-Bx) and miraxanthin V (dopamine-Bx) are less capable of reducing 1,1-diphenyl-2-picrylhydrazyl radicals (DPPH) than ascorbic acid [32]. They prepared two isomers of lysine-betaxanthin that were characterized only by mass spectrometry and that have been described by other authors without further characterization [17,27,32,33,34,35]. Gandía-Herrero’s group produced betaxanthins on a 100-mg scale using the DODA of *G. diazotrophicus* [36]. They prepared nine amino acid betaxanthins and the pseudo-natural pyrrolidine-betaxanthin and investigated their antioxidant and radical-scavenging properties in vitro, as well as their effect on the lifespan of *Caenorhabditis elegans* [37]. Indicaxanthin, phenylalanine-betaxanthin, and dopaxanthin increased the lifetime of the worm up to 17%, an effect that was linked to the activation of the DAF-16/FOXO and SKN-1/Nrf2 transcription factors but did not follow the antioxidant performance trend found in vitro.

Here, we provide a molecular explanation of how substituents at the α-C of the amino acids influence the antioxidant and redox properties of amino acid betaxanthins and why these biocompatible natural pigments are efficient antioxidants. Twenty-four betaxanthins were semisynthesized, including the derivatives of all twenty proteinogenic α-amino acids and the betaxanthins of l-DOPA, dopamine, and pyrrolidine. The cysteine-betaxanthin (Cys-Bx) was characterized for the first time and has the lowest antioxidant capacity among all compounds studied, despite the high antioxidant potential of cysteine and thiols in general. The redox properties and pH-dependent antioxidant capacity of these amino acid betaxanthins were analyzed in terms of their structural features and the combination of multiple proton-coupled electron transfer (PCET)-active sites increase their antioxidant capacity, regardless of whether the betaxanthin is phenolic or not.

## 2. Materials and Methods

### 2.1. General

Commercial chemicals were purchased from Sigma-Aldrich (Milwaukee, St. Louis, MO, USA). Solutions were prepared using deionized water (18.2 MΩ∙cm at 25 °C, total organic carbon (TOC) ≤ 4 parts per billion, Milli-Q, Millipore, MA, USA). Results are expressed as mean ± standard deviation of triplicate experiments.

### 2.2. Obtaining of Betalamic Acid

Betalamic acid was extracted from red beetroot juice as described previously [27,38]. Briefly, beetroot juice (0.5 L) was centrifuged (3000× *g*, 10 min, 25 °C), filtered, and the supernatant was alkalinized to pH 11.4 with concentrated NH_4_OH. The mixture was kept under stirring until its color changed from magenta to dark yellow/brown (ca. 30 min, 25 °C), cooled with an ice bath, and then acidified with concentrated HCl until pH 2.0 was reached. Betalamic acid was extracted with ethyl acetate (2 × 50 mL) and the resulting bright yellow solution was kept at −78 °C until a white precipitate containing mainly hydrated ammonium chloride was formed. The solid was removed by vacuum filtration and the resulting solution of HBt in ethyl acetate was concentrated. Immediately before use, betalamic acid was transferred to an aqueous solution of NH_4_OH pH 11.0.

### 2.3. Semisynthesis of Betaxanthins

To a round-bottom flask equipped with magnetic stirring, a solution of betalamic acid in aqueous NH_4_OH pH 11.0 (20 mL, 6 × 10^−4^ mol L^−1^) and either the neat amino acid or amine (10–100 equiv.) were added. The reaction was kept at 25 °C until the complete depletion of the absorption band of betalamic acid with a maximum at 424 nm. The solution was cooled with an ice bath and HCl (conc.) was added dropwise until pH 5.0 was reached. The solution was allowed to reach room temperature (25 °C) for 30 min and the volume was reduced to 2–4 mL by freeze-drying. The product was purified through semi-preparative reversed phase high performance liquid chromatography (RP-HPLC, Supelco Ascentis C18 25 cm × 1 cm, 5 µm); the conditions were as follows. A: water/HCO_2_H (0.05% *v*/*v*), B: MeCN/HCO_2_H (0.05% *v*/*v*); flow rate: 2 mL min^−1^; 2% B (Asn-Bx), 10% B (Arg-Bx, DA-Bx, l-DOPA-Bx, Pro-Bx, Tyr-Bx), 2–15% B in 30 min (His-Bx), 2–30% B in 30 min (Ala-Bx, Asp-Bx, Gln-Bx, Glu-Bx, Gly-Bx, Ile-Bx, Leu-Bx, Lys-Bx, Met-Bx, Ser-Bx, Thr-Bx, Val-Bx), and 10–60% B in 30 min (Phe-Bx, Tyr-Bx). Fractions containing the betaxanthin were collected, combined and the pH was adjusted to 5.0 by adding aqueous NH_4_OH (0.1 mol L^−1^). The resulting solution was freeze-dried, and the solid product was kept in the dark at −20 °C. Each betaxanthin was repurified by gel permeation chromatography (Sephadex LH-20/water) immediately before use and the concentration of the stock solution in aqueous media was determined by UV-vis absorption spectrophotometry using a molar absorption coefficient at 480 nm of 4.8 × 10^4^ L mol^−1^ cm^−1^ [27,39].

### 2.4. High-Performance Liquid Chromatography Coupled to Mass Spectrometry

HPLC-ESI-qTOF-MS/MS data were acquired using a Shimadzu 20A liquid chromatograph equipped with an SPD-20A PDA detector and coupled with a Bruker Daltonics MAXIS 3G ESI-qTOF mass spectrometer, operating in the positive ion mode. The betaxanthins were analyzed with an Ascentis C18 column (5 μm, 250 mm × 4.6 mm, Supelco) at 1 mL min^−1^ and 25 °C under a gradient from 5% to 65% B in 20 min [A: water/HCO_2_H (0.05% *v*/*v*), B: MeCN/HCO_2_H (0.05% *v*/*v*)].

### 2.5. Nuclear Magnetic Resonance Spectroscopy

^1^H NMR spectra of betaxanthins were recorded using a Bruker Avance III 500 spectrometer, operating at 500 MHz at 298 K. The zgpr pulse program (presaturation) was used to suppress the residual water signal. Solutions were prepared in D_2_O (500 μL, 0.3 mg mL^−1^) immediately before spectral acquisition. Chemical shifts are reported as δ values (ppm) referenced to trimethylsilyl propionate (TSP) or D_2_O. All spectra were processed using the software MestReNova (v. 11.0.4, 2017, Mestrelab Research S.L., Santiago de Compostela, Spain).

### 2.6. Trolox Equivalent Antioxidant Capacity

#### 2.6.1. ABTS Assay

The TEAC/ABTS^•+^ assay was performed according to the protocol of Re et al. [40]. A stock solution of ABTS^•+^/ABTS in water was prepared via partial oxidation of ABTS [2,2′-azinobis-(3-ethyl-benzothiazoline-6-sulfonic acid), 7 mmol L^−1^] by potassium persulfate (2.45 mmol L^−1^) in the dark at room temperature for 16 h. The stock solution of ABTS^•+^/ABTS was diluted in phosphate buffer pH 7.0 (0.1 mol L^−1^) or acetate buffer pH 3.6 (0.3 mol L^−1^) to an absorbance of 0.7 (46.7 μmol L^−1^ ABTS^•+^) at 734 nm. The kinetics of bleaching of ABTS^•+^ after the addition of antioxidant were monitored by a change in the absorption at 734 nm for 6 min (ΔA) at 25 °C. The slope of the linear correlation between ΔA and the concentration of antioxidant, α, is a measure of antioxidant capacity. Consequently, the α_sample_/α_Trolox_ ratio is the Trolox equivalent antioxidant capacity, TEAC_ABTS_.

#### 2.6.2. FRAP Method

The ferric-reducing antioxidant power (FRAP) was determined using the protocol developed by Benzie and Strain [41]. The FRAP working solution was prepared by combining 2.5 mL of a 10 mmol L^−1^ solution of TPTZ (2,4,6-tripyridyl-*s*-triazine) in 40 mmol L^−1^ aqueous HCl, 2.5 mL of a 20 mmol L^−1^ solution of FeCl_3_⸱6H_2_O in water, and 25 mL of 0.3 mmol L^−1^ acetate buffer pH 3.6. The assay was performed by measuring the change in the absorption at 593 nm for 30 min (ΔA) at 37 °C due to the reduction of the ferric-tripyridyltriazine (Fe(III)-TPTZ) complex to its ferrous form (Fe(II)-TPTZ) upon the addition of the antioxidant. The slope (*α*) of the linear correlation between ΔA and the antioxidant concentration was measured. The antioxidant capacity was calculated as the ratio between the value of *α* for the sample and for the antioxidant standard Trolox (1–5 µmol L^−1^ concentration range), *α*_Sample_/*α*_Trolox_, and is reported as TEAC_FRAP_.

### 2.7. Cyclic Voltammetry

Cyclic voltammograms were acquired at 25 °C using a Metrohm Autolab PGSTAT101 potentiostat/galvanostat, controlled using the proprietary software NOVA. A screen-printed carbon working electrode (Metrohm, Herisau, Switzerland; DropSens DRP-110; 6.1208.110) with a carbon auxiliary electrode and a silver reference electrode was used; potential range: −0.4 to 1.0 V, scan rate: 50 mV s^−1^, analyte concentration: 1 mmol L^−1^ in aqueous KCl (0.1 mol L^−1^) at pH 7.0.

### 2.8. Computational Methods

The structures of the fully protonated forms of the betaxanthins were arbitrarily built in their *trans* and (*S*)-C6 configurations and submitted to a conformational search using quantum chemical GBSA/GFN2–xTB methods, as implemented in the Conformer–Rotamer Ensemble Sampling Tool (CREST, Bonn, Germany) [42,43]. The lowest energy conformers of each betaxanthin, as well as their 1e^–^-oxidized and deprotonated forms, were submitted to geometry optimization in water at the SMD/M06-2X/6-31+G(d,p) level of theory [44,45,46] using the Gaussian16 rev. C.01 program suite [47]. Stationary points were characterized as minima based on vibrational analysis. Partial charges of the nitrogen atoms were calculated according to the Merz–Kollman–Singh (MKS) scheme constrained to reproduce the dipole moment (μ). All structures and surfaces were rendered by the software Chemcraft (1.8 build 622). Marvin Sketch (21.17.0, ChemAxon, Budapest, Hungary) was used for the initial estimation of p*K*_a_ values. Genetic functions were created constraining the maximum number of independent variables to three, restricting the model to linear correlations, and considering up to 5000 generations using the Friedman Lack-of-Fit as a scoring function using the Matlab software (2022b, Mathworks, Natick, MA, USA) [48]. Matched molecular pair analysis (MMPA) was performed to find activity cliffs according to the procedure described by Hussain and Rea [49] and considering a single cut.

## 3. Results

### 3.1. Semisynthesis of Betaxanthins

**Figure 1:**
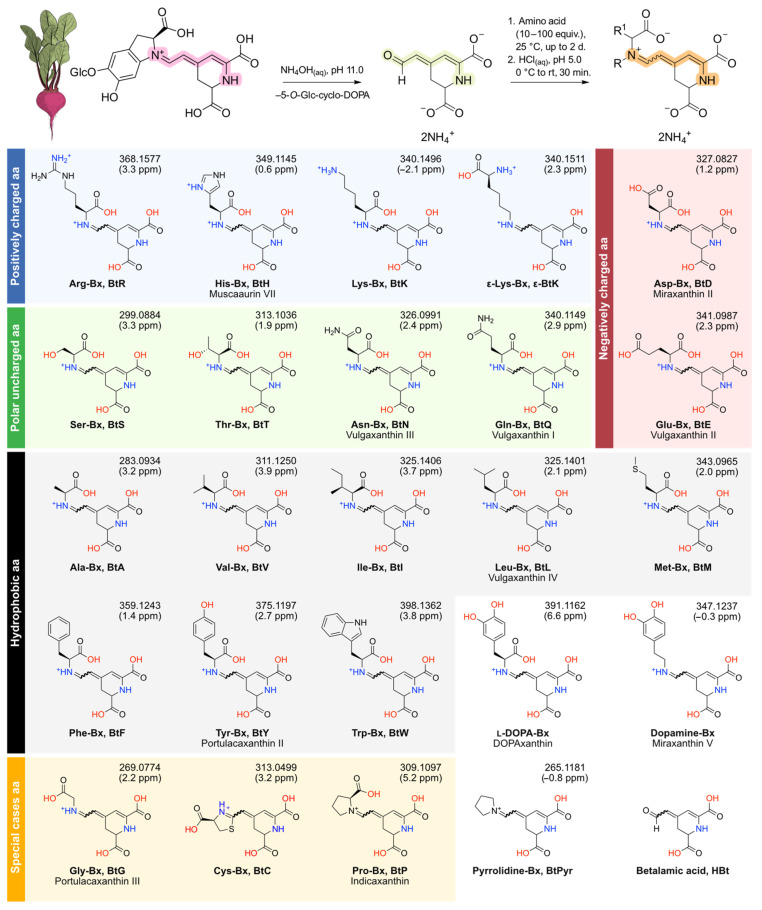
Semisynthesis of amino acid betaxanthins and chemical structures of the betaxanthins prepared and their common precursor, betalamic acid. The betaxanthins are in the form of ammonium salts before purification. Compounds are classified according to the properties of their precursor amino acids (aa) and are presented in the fully protonated form for clarity. The wavy bond indicates that both diastereomers are formed. R is the substituent at the amino group of the aa or hydrogen; R^1^ represents the side chain. Numbers at the top of each structure are the observed *m/z* and the difference between the calculated and observed *m*/*z* values, in parts per million (ppm). Mass spectra and fragmentation patterns for all compounds are given in Figures S2–S25.

Twenty-four betaxanthins were prepared by coupling betalamic acid (HBt) and excess l-amino acids (aa) or amines in aqueous ammonia solution at pH 11.0 and 25 °C (Figure 1). All reactions were carried out in the dark and after HBt had been consumed, as inferred by the disappearance of its absorption band centered at 424 nm, the solution was cooled with an ice bath and the pH was adjusted to 5.0. Next, the cooling system was removed, and the solution was let to reach room temperature (25 °C) for 30 min. Both l-proline and pyrrolidine react quickly with HBt and were prepared within 1 h using a 10 equiv. excess of the amino acid/amine. Betaxanthins derived from primary amino acids/amines required 100 equiv. of precursor and the reaction took up to 2 d to complete. For the betaxanthins of dopamine, l-DOPA and l-tyrosine, the reaction was performed under nitrogen atmosphere to retard oxidation. Products were purified by semipreparative reversed-phase HPLC followed by gel filtration on Sephadex LH-20 in water. Due to their instability in solution, products were characterized by HPLC-ESI-qTOF-MS/MS and, in the case of Cys-Bx, Lys-Bx, and ε-Lys-Bx, by ^1^H NMR spectroscopy also. When minor chromatographic signals indicated the presence of stereoisomers in solution, only the products with the highest absorption at 480 nm were collected during chromatographic purification (Figure S1) and, hence, the configuration of some double bonds and of the C6 of the compounds presented in Figure 1 are undefined. Samples were always protected from light and stored as a solid at −20 °C until use. Reaction yields were determined spectrophotometrically and are below 5% in all cases.

#### 3.1.1. The Elusive Betaxanthin of l-Cysteine

**Figure 2:**
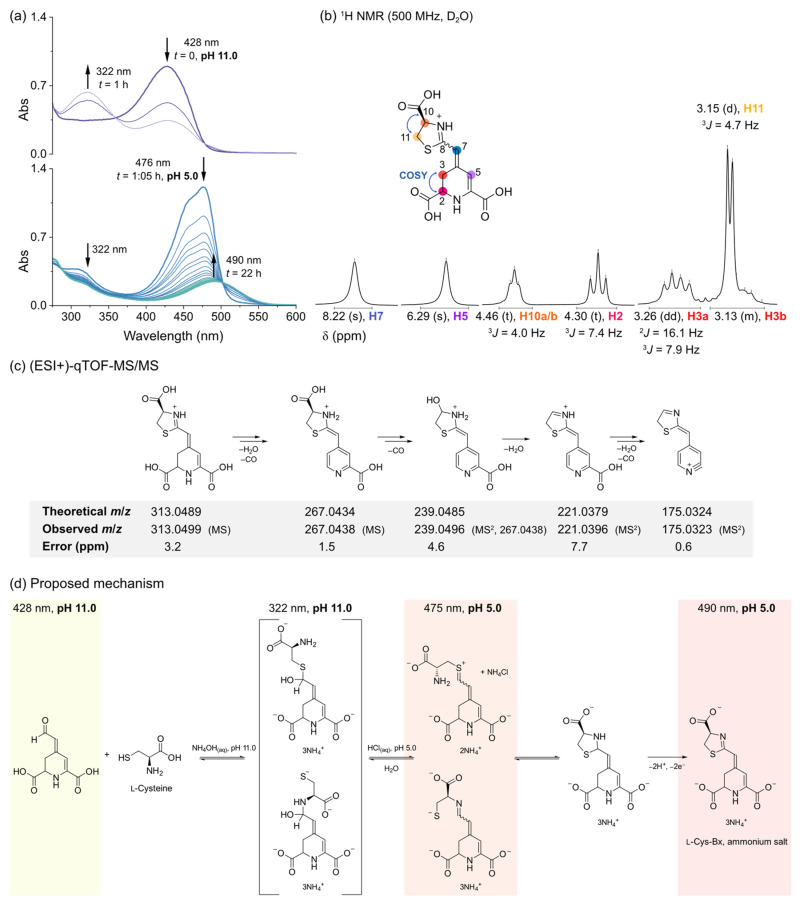
Characterization of Cys-Bx. (**a**) UV-Vis absorption spectra over time. (**b**) ^1^H NMR analysis. Blue arrowed curves indicate couplings found in ^1^H,^1^H COSY analysis (Figure S27). (**c**) Mass fragmentation pattern of Cys-Bx; raw MS data are presented in Figure S6. (**d**) Proposed mechanism for the formation of Cys-Bx in solution.

Attempts to use the semisynthesis protocol to prepare the l-cysteine betaxanthin under alkaline conditions led to species with an absorption maximum (λ^Abs^) at 322 nm (Figure 2a). The acidification of the reaction media to pH 5.0 with aqueous HCl immediately resulted in depletion of the band centered at around 322 nm and the appearance of a transient species showing an absorption spectrum similar to that of amino acid betaxanthins and with an λ^Abs^ at 476 nm. Over 22 h, the absorption at 476 nm decreased with the appearance of an absorption band centered at 490 nm (Figure 2a).

Analysis of Cys-Bx by ^1^H NMR spectroscopy revealed that the signal corresponding to the H8 of amino acid betaxanthins was absent (Figure 2b, Figures S26 and S27). The H7 appears as a signal with singlet multiplicity and is deshielded in ca. 2 ppm compared to those of proline-betaxanthin and dopamine-betaxanthin, for example, in which H7 is a doublet coupled with H8 (^3^*J*_H7,H8_~12 Hz) [50]. Nevertheless, the ABX system formed between H2 and H3a/H3b and the signal corresponding to H5 show the typical multiplicities and chemical shifts expected for betalains [50,51]. The triplet (δ = 4.46 ppm) and the doublet (δ = 3.15 ppm) signals were attributed to a 2-thiazoline ring (Figure 2b), in agreement with the results obtained with high resolution tandem mass spectrometry (Figure 2c). The *m*/*z* found is compatible with the presence of a 2-thiazoline instead of the expected betaxanthin of l-cysteine or its pyridine analogue (neobetalain). Fragmentation occurs mainly via elimination of CO and water and corroborates the proposed structure for Cys-Bx (Figure 2c). We could not perform ^13^C NMR experiments due to the limited amounts of compound produced and their poor persistence in water. Considering all the evidence, we tentatively attributed the species with λ^Abs^ at 322 nm as a tetrahedral intermediate formed by the attack of the thiolate nucleophile (and/or, less likely, the amino group of cysteine) to betalamic acid, and the species absorbing at 476 nm as the expected betaxanthin of cysteine or its sulfonium isomer. The spontaneous cyclization of either species leads to a thiazolidine ring that is oxidized to the Cys-Bx bearing the 2-thiazoline ring (Figure 2d). This is the first report on the structure of Cys-Bx and the first non-iminic betalain derived from amino acids.

#### 3.1.2. The Two Betaxanthins of l-Lysine

**Figure 3:**
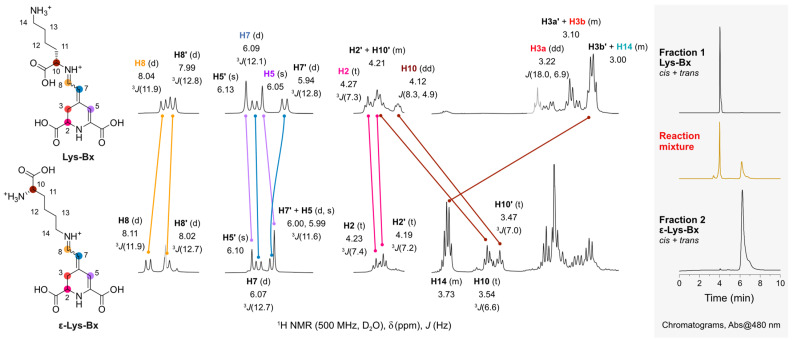
^1^H NMR analysis of Lys-Bx and ε-Lys-Bx. Atom numbering in the amino acid chain was kept identical for clarity. Signals in grey are of impurities. Full spectra are presented in Figures S28 and S29.

The chromatographic analysis of the reaction between betalamic acid and l-lysine revealed four peaks with an *m*/*z* compatible to that of isomers of Lys-Bx. We managed to isolate two fractions enriched with the compounds with a retention time of 4.1–4.2 min (fraction 1) or 6.1–7.2 min (fraction 2) by using HPLC in the semipreparative scale. Both fractions were freeze-dried, resuspended in D_2_O, and submitted to ^1^H NMR spectroscopic analysis (Figure 3). In both samples, we found duplicated signals indicating the presence of stereoisomers, most likely *E*/*Z* diastereomers that cannot be resolved by non-chiral chromatographic methods [52]. The chemical shifts (δ) of the signals corresponding to H5, H7, and H8 in the compounds in both fractions are similar and agree with the typical values found for natural betalains. The H10 at the α-C, however, is largely deshielded (Δδ~0.8 ppm) in the isomers in fraction 1 compared to those in fraction 2, suggesting that they are enriched with the products of the attack of the α- and ε-amino group, respectively, to betalamic acid. The δ of H10 in Lys-Bx is similar to the value reported for proline-betaxanthin [50]. For ε-Lys-Bx, the δ of H14 is compatible with that determined for the C*H*_2_ directly attached to the imino group of the dopamine-betaxanthin (3.60 ppm), which lacks the α-carboxylic acid moiety [50]. The fact that the signal corresponding to the H14, located at the side chain of Lys, is far more shielded in the isomers of fraction 1 than in fraction 2 supports our conclusion. ^1^H NMR analysis of the isomers of Lys-Bx was possible since only minor impurities were present. Unfortunately, we could not attribute the signals corresponding to H11–H13 in the isomers in fraction 2 unequivocally. Nevertheless, the antioxidant and redox properties of both fractions were characterized.

### 3.2. Antioxidant Capacity and Redox Potentials of Amino Acid Betaxanthins

The capacity of the amino acid betaxanthins in undergoing single electron transfer (SET) and proton-coupled electron transfer (PCET) was investigated by determining their antioxidant capacities using ABTS and FRAP colorimetric assays. The ABTS assay is based on the capacity of an antioxidant to reduce the bluish-green non-biological radical cation of 2,2′-azino-bis(3-ethylbenzthiazoline-6-sulfonic acid) (ABTS^•+^) via both SET and PCET from the antioxidant to ABTS^•+^ and depleting its absorption signal at 734 nm. The FRAP assay, on the other hand, is based on the 1e^–^-reduction of a Fe^3+^ complex in acidic media (via SET) to produce a deep blue ferrous product. Results are presented as Trolox equivalent antioxidant capacity (TEAC) for comparison and, as the FRAP assay requires acidic media at pH 3.6 to solubilize the Fe^2+^ product, we measured the TEAC_ABTS_ at both pH 3.6 and 7.0. Ascorbic acid (AscH) was used as an additional reference antioxidant and control experiments included betalamic acid, dopamine-betaxanthin, l-DOPA-betaxanthin and pyrrolidine-betaxanthin. Despite their natural occurrence, the antioxidant capacity of most amino acid betalains has not been studied before, and the results available in the literature for some of these compounds are hardly comparable since they remount to several groups using different analytical methods [37,53,54,55].

**Figure 4:**
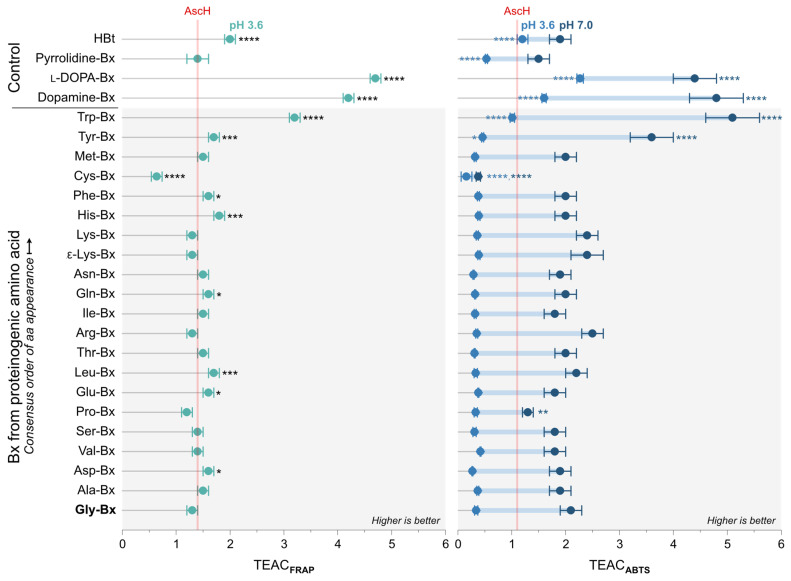
Trolox equivalent antioxidant capacity (TEAC) of betalamic acid and betaxanthins derived from l-amino acids, dopamine, and pyrrolidine determined by the FRAP method at pH 3.6 and by the ABTS assay at pH 3.6 and 7.0. Raw data are presented in Figures S30–S33. Asterisks indicate values that are different from those of Gly-Bx (ANOVA-Tukey test, 95% confidence interval, * *p* < 0.05, ** *p* < 0.01, *** *p* < 0.001, **** *p* < 0.0001, *N* = 3).

The results of the FRAP assay show that at pH 3.6, most betaxanthins have a TEAC_FRAP_ lower than 2, which is similar to the capacity of AscH (Figure 4). Exceptions are Cys-Bx, which showed the lowest capacity, and tryptophan-betaxanthin (3.2 ± 0.1), dopamine-betaxanthin (4.2 ± 0.1), and l-DOPA-betaxanthin (4.7 ± 0.1) that presented the highest TEAC_FRAP_ values. Tyrosine-betaxanthin has a TEAC_FRAP_ of 1.7 ± 0.1, indicating that the phenolic group does not necessarily increase the TEAC_FRAP_ compared to non-phenolic betaxanthins such as His-Bx, for example. TEAC_ABTS_ results obtained at pH 3.6 are lower than unity, meaning that in acidic media, these betaxanthins scavenge less ABTS^•+^ than Trolox and AscH. Again, Cys-Bx has the lowest TEAC_ABTS,_ and Tyr-Bx (0.46 ± 0.02) did not outperform non-phenolic betaxanthins. At pH 7.0, however, all betaxanthins, become better radical scavengers than AscH and Trolox, except for cysteine-betaxanthin. Tyrosine-betaxanthin (3.6 ± 0.4) joins the group of high-capacity antioxidants together with dopamine-betaxanthin, (4.8 ± 0.5), l-DOPA-betaxanthin (4.4 ± 0.4), and Trp-Bx (5.1 ± 0.5).

**Figure 5:**
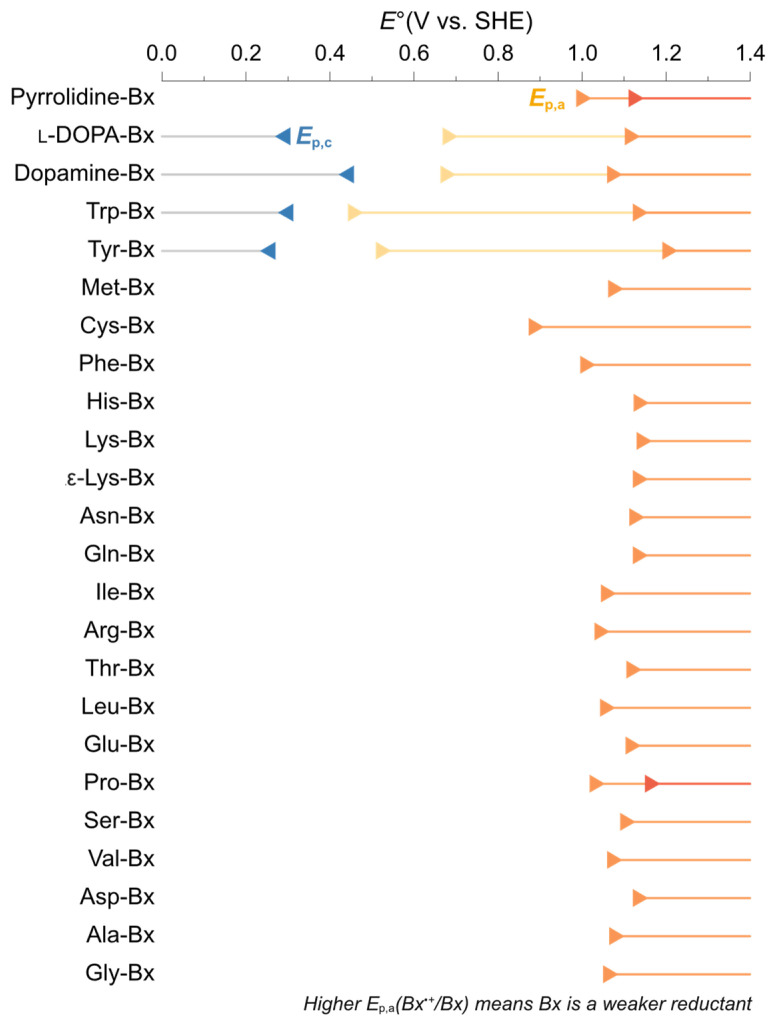
Peak potentials of betaxanthins derived from l-amino acids, dopamine, and pyrrolidine determined by cyclic voltammetry. Cathodic (◄) and anodic (►) potentials were determined using screen printed vitreous carbon electrodes against an Ag/AgCl reference electrode and a carbon auxiliary electrode; potential range: −0.4 to 1.0 V, scan rate: 50 mV s^−1^, betaxanthin concentration: 1 mmol L^−1^ in aqueous KCl (0.1 mol L^−1^) at pH 7.0.

Information on the redox potentials of betaxanthins is required for the in-depth analysis of their radical scavenging properties. Therefore, cyclic voltammetry was carried out in aqueous KCl 0.1 mol L^−1^ at pH 7.0 aiming to determine formal potentials (Figure S34). All betaxanthins showed at least one anodic wave, with peak potentials ranging between 0.4 and 1.2 V vs. SHE (Figure 5). Unfortunately, most betaxanthin studied depict an irreversible chemical reaction following the electrochemical process, making it difficult to determine their half-wave potentials from the anodic and cathodic peak potentials (*E*_p,a_, *E*_p,c_, respectively) [56]. Inflection-point potentials (*E*_i_) have been used to approximate formal redox potentials when irreversibility prevails [57] and are presented in Table S1 for reference. Dopamine-betaxanthin, l-DOPA-betaxanthin, tryptophan-betaxanthin and tyrosine-betaxanthin show reversible waves and lower *E*_p,a_ values compared to other betaxanthins. The *E*_p,a_ of Cys-Bx is at around 0.9 V and its current response is much lower than that of other betaxanthins, suggesting lower ionization [58] (Figure S34).

The effect of pH on the electrochemical properties of Pro-Bx was evaluated by square wave voltammetry using a boron-doped diamond electrode (Figure S35). The iminium nature of Pro-Bx enables the investigation of the influence of other ionizable portions, especially N1*H*, on the redox potential of amino acid betaxanthins. The potential required for the oxidation of Pro-Bx is clearly increased at around pH 6.0, while the intensity of peak current decreases as the medium becomes more alkaline, reaching a value close to zero at this pH.

### 3.3. Insight on the Structural Features of Betaxanthins

**Figure 6:**
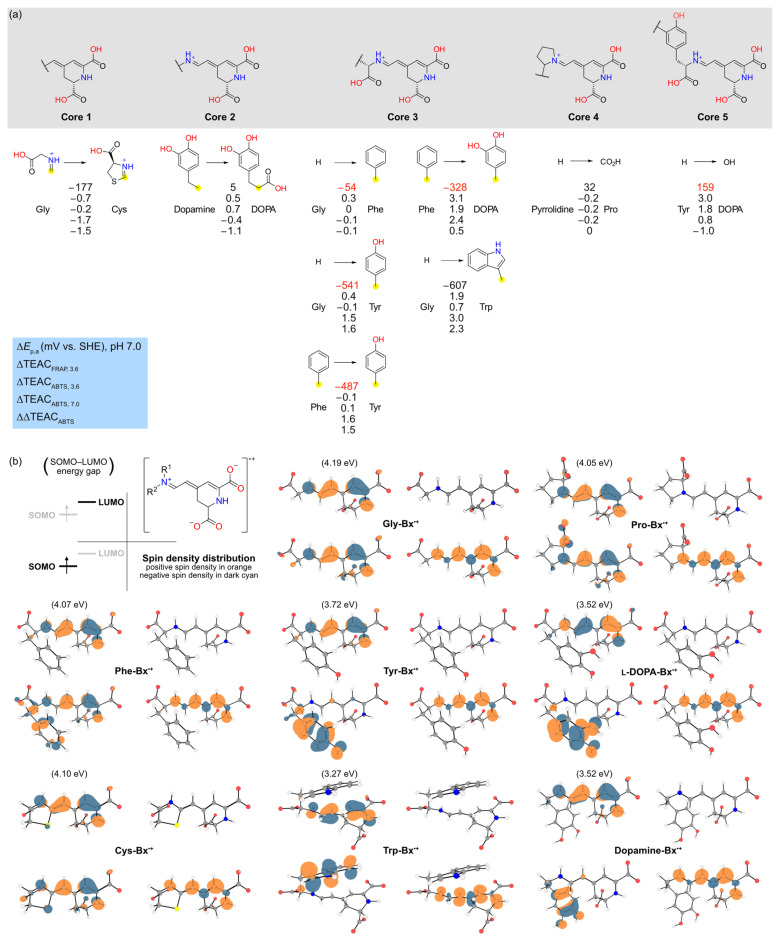
Matched molecular pair analysis (MMPA) and quantum chemical calculations. (**a**) MMPA with five cores and a single cut; attachment points with open valence are highlighted in yellow. Numbers are the experimental parameters indicated in the blue legend. (**b**) Structure, spin density distribution (isovalue 0.005) and frontier molecular orbitals (isovalue 0.05) of the radical cations of selected betaxanthins (doublets, referred to as radical cations for simplicity since the formal charge varies). Description of values and surfaces are presented in the legend in the up-left corner.

Matched molecular pair analysis (MMPA) was performed to find activity cliffs in the antioxidant capacity of amino acid betaxanthins [49]. The threshold used for the analysis was defined considering the values of *E*_p,a_ and TEAC that are statistically different from those of Gly-Bx (Figure 4), although additional comparisons were performed in order to establish structure–property relationships. We selected five main structural cores (identical structural portions that are not expected to affect the results when comparing a set of betaxanthins) and evaluated the effect of replacing the indicated substituents on the observed change in the anodic peak potential at pH 7.0 (Δ*E*_p,a_), TEAC_FRAP_ at pH 3.6 (ΔTEAC_FRAP, 3.6_), TEAC_ABTS_ at pH 3.6 (ΔTEAC_ABTS, 3.6_), TEAC_ABTS_ at pH 7.0 (ΔTEAC_ABTS, 7.0_), and variation of TEAC_ABTS_ upon the increase in pH from 3.6 to 7.0 (ΔΔTEAC_ABTS_) (Figure 6a).

The substitution of H by a CO_2_H at the α-C of the amino acid portion has modest effects in all parameters, as inferred from comparing the pairs dopamine-Bx/l-DOPA-Bx (core 2) and pyrrolidine-Bx/Pro-Bx (core 4). Core 3 includes the imino acid portion of primary amino acid betaxanthins, enabling the evaluation of the effects of the side groups at the α-C. The exchange of H by a phenyl (Ph) group has no effect on the peak potential and antioxidant capacity of amino acid betaxanthins. However, when H or Ph is substituted by a phenol moiety, a steep decrease of approximately 500 mV in the anodic potential is observed, followed by an increase in TEAC at pH 7.0, but not at pH 3.6. Replacement of Ph by a catechol (Phe-Bx vs. l-DOPA-Bx) has a much lower effect on *E*_p,a_ (−328 mV change) than the variation for Ph versus phenol (−487 mV), which agrees with the increase in *E*_p,a_ observed when phenol is replaced by catechol (Tyr-Bx vs. l-DOPA-Bx, core 5). Replacement of the glycinyl moiety by a (*R*)-4-carboxy-4,5-dihydro-2λ^3^-thiazol-3-ium group reduces the value of all parameters (core 1). The largest effect on the *E*_p,a_, (−607 mV) is observed when H is replaced by an indolyl group at core 3 (Gly-Bx vs. Trp-Bx). This modification also results in the highest variation in TEAC_ABTS, 7.0_ and a noticeable change in TEAC_FRAP, 3.6_ and TEAC_ABTS, 3.6_.

The lowest-energy conformers of the betaxanthins used in MMPA underwent one-electron oxidation and their geometry was optimized in water (Figure 6b). Analysis of the spin density distribution reveals that in all cases, the total difference between α and β electrons is mostly located in the 1,7-diazaheptamethinium of the betaxanthins, except for Cys-Bx in which a node is found at the C8. The singly occupied molecular orbital (SOMO) and the lowest unoccupied molecular orbital (LUMO) of most betaxanthins are in the 1,7-diazaheptamethinium (1,7-dAhM) of the betaxanthins, evidencing two enamine systems separated by a node at the C4, which contradicts the conventional interpretation based on resonance structures. For the radical cations of Tyr-Bx, l-DOPA-Bx, dopamine-Bx, and Trp-Bx, however, the SOMO is located at the substituent at the α-C of the amino acid portion, while the LUMO is still located in the 1,7-dAhM system. Under the same analysis conditions, Phe-Bx^•+^ is an intermediate case, in which the SOMO is delocalized throughout the whole molecule (although more evident in the 1,7-dAhM). The SOMO–LUMO gap was calculated and is around 4.1 for the radical cations of the betaxanthins derived from Gly, Pro, Phe, and Cys. The betaxanthin radical cation derived from Tyr, l-DOPA and dopamine has a gap of approximately 3.6, while the value for Trp-Bx^•+^ is 3.3 eV.

## 4. Discussion

**Betaxanthins can be semisynthesized in a counterintuitive manner.** Betalamic acid is obtained by alkaline hydrolysis of the betalains, mainly betanin and isobetanin, in beetroot juice [27]. Therefore, the use of alkaline conditions for the semisynthesis of amino acid betaxanthins from betalamic acid may seem illogical. However, under the conditions reported here, the deprotonation of the ammonium groups of amino acids (p*K*_a_~9.5) promotes the 1,2-nucleophilic addition of the amino group to the aldehyde function of betalamic acid, producing betaxanthins quantitatively, as inferred by TLC analysis. Hence, we conclude that the poor isolated yields observed for the semisynthesis of amino acid betaxanthins are due to decomposition during purification.

Betalamic acid reacts much faster with l-proline or pyrrolidine than with the other amino acids and primary amines, for which 10 times more nucleophile and up to 2 days were used. This is due to the higher nucleophilicity of secondary amines compared to their primary counterparts, as evidenced by the Mayr’s nucleophilicity parameter, *N*, e.g., of Pro (18.1), Pyr (17.2), Phe (14.1), and Thr (12.7) [59,60]. The procedure reported here avoids the use of a large excess (>500 equiv.) of amino acid/amine and acid is used only to bring the solution to pH 5.0 to avoid betaxanthin decomposition.

**Obtaining enantiopure betaxanthins in water is challenging**. The semisynthesis of betaxanthins is usually performed in ethyl acetate or water as solvents. In ethyl acetate, the reaction requires acid-catalysis and *p*-toluenesulfonic acid has been used as catalyst, which has the additional advantage of inducing the precipitation of the product [27,38,61,62,63]. Nevertheless, the reaction in ethyl acetate is limited to lipophilic reactants, which is not the case of most amino acids. As betalains have, in general, been shown to decompose in acidic aqueous media, their semisynthesis in water is often performed at a pH higher than 3.0 [64]. The resolution of betaxanthins [(*S*)-C6] and their *iso* stereoisomers ((*R*)-C6), as well as the isolation or synthesis of a single *cis*/*trans* diastereoisomer, is unproductive because betaxanthins and other imines isomerize in aqueous solution [65,66,67]. Therefore, like all other studies carried out using semisynthetic betaxanthins, the results reported here refer to a mixture of at least two stereoisomers of each betaxanthin.

**Amino acid betaxanthins are high-capacity antioxidants.** Antioxidant capacity and activity assays performed in vitro contribute to the overall understanding of the mechanisms of antioxidant action and to establishing structure–property relationships, although their results do not necessarily reflect or explain the complex behavior and effectiveness of antioxidants in vivo [68]. Any antioxidant assay can, in principle, be used to determine how many radicals or electrons have been scavenged per molecule of antioxidant (capacity) and/or how fast (reactivity/activity) this reaction is, providing their limitations are known and the results are not overinterpreted or extrapolated to complex biological matrices without further evidence [69]. Results obtained with SET-based methods have been considered to have a poor correlation with those obtained by methods relying on other antioxidant mechanisms, such as PCET, and the combination of antioxidant assays have been suggested as a form of accessing different aspects of the antioxidant properties of a given reductant [68,70]. Therefore, we combine cyclic voltammetry and FRAP measurements, whose results are related to electron transfer processes, with the ABTS assay that provides information on SET/PCET-mediated reductions to investigate how the structure of the precursor amino acid affects the antioxidant properties of betaxanthins.

Although amino acid betaxanthins have been found in vegetables and fungi [16,17,20,34,53,71] and the antioxidant capacity of some of them have been described previously [32,37,53,72], the complete study of betaxanthins derived from all proteinogenic amino acids, except selenocysteine, is reported for the first time. The fact that all experiments have been performed using compounds prepared, purified, and studied in the same way increase the confidence for comparing the results obtained. Our results on Pro-Bx (indicaxanthin) agree with reports showing that, despite being a well-known antioxidant and anti-inflammatory agent in vivo [73,74,75], Pro-Bx does not have an exceptional antioxidant capacity [55,72], and is lower than that of vulgaxanthin I (Gln-Bx) [72], which is abundant in Golden beetroots [76]. These results have been rationalized by considering that the presence of a positively charged iminium group in indicaxanthin decreases its antioxidant capacity [53,72], but this interpretation implies that other more negatively charged betaxanthins may have even better antioxidant properties and/or that the iminium portion of Pro-Bx is important for its biological properties.

Considering chemical arguments, a good chain-breaking non-enzymatic antioxidant must react with radicals quickly and in a spontaneous manner, and the products of this reaction must terminate the radical chain or slow it down significantly. For example, one-electron oxidation of ascorbic acid produces the ascorbate radical that, upon a second 1e^–^-oxidation gives the stable dehydroascorbic acid. Trolox 1e^–^-oxidation produces a resonance stabilized radical that, upon further 1e^–^-oxidation yields a closed-shell quinone. Therefore, it is apparently easy to explain why Trolox can scavenge two radicals per molecule, as determined by many independent groups after the seminal work of Ingold and collaborators [77], but it is hard to justify why, in some cases, ascorbic acid has a lower radical scavenging capacity than Trolox. For these standards and other antioxidants having acidic O–H, N–H, and/or S–H groups, increasing the pH promotes deprotonation and, consequently, oxidation tends to occur in a more spontaneous manner [78], illustrating the importance of the relationship between proton and electron transfer for the radical scavenging properties of small organic molecules [79]. This simple rationalization does not apply when the number of radicals scavenged by a single molecule of antioxidant becomes too high for having a straightforward physical meaning, suggesting the occurrence of complex processes. We show that most substituents at the α-C were unable to modulate the redox and antioxidant properties of betaxanthins, despite their polarity and charge, as shown for the two stereoisomers of Lys-Bx, whose antioxidant and redox properties are identical to those of Gly-Bx, regardless of which amino group of Lys is attached to the betaxanthin scaffold. Marked exceptions are the indole moiety of Trp, and the catechol groups of dopamine and l-DOPA. The phenol substituent of Tyr, however, influences the TEAC at pH 7.0 but not under acidic conditions, suggesting that deprotonation enhances its antioxidant capacity. Last, the pH effect on the peak potential and peak current of Pro-Bx suggests the existence of a p*K*_a_ of around 6, a fact that cannot be easily rationalized considering the estimated p*K*_a_s of betaxanthins (Table S2), but that may are linked to the p*K*_a_ of 6.8 found for betalamic acid [80].

**Both indole (N–H) and catechol (O–H) moieties enhance the antioxidant capacity of betaxanthins.** Although many endogenous antioxidants contain nitrogen and sulfur moieties, most natural and artificial exogenous antioxidants are phenols [78]. This notion is evidenced by the successful use of anthocyanins, natural polyphenolic compounds, as additives in food and cosmetics, as well as their use as phytomedicines [81,82]. Consumers consider that natural ingredients promote health benefits; hence, the identification of biocompatible non-phenolic natural antioxidants has gained increasing economic importance [83,84,85]. Betalains emerged as redox mediators of natural origin [61,62,86,87], but they are still less understood than other classes of natural antioxidants [52]. The 1,7-diazaheptamethinium system of betaxanthins has been shown to stabilize radicals by resonance [15,88,89]. However, the group attached to the N9 of the 1,7-dAhM group can affect the antioxidant capacity of betaxanthins in either a positive or negative manner. To investigate the antioxidant capacity of betaxanthins using quantum chemical calculations, we assumed the analysis of the spin density distribution, as well as the properties of the frontier orbitals of the radical cation of those examples in which the substituent caused major variations using Gly-Bx^•+^ as a reference. In fact, the spin density of betaxanthins show TEAC and *E*_p,a_ values similar to those of Gly-Bx and highlight that, considering all orbitals and the population of α and β electrons, the highest spin density is located within the 1,7-dAhM system. However, a comparison of SOMO and LUMO revealed that those betaxanthins with higher antioxidant capacity and lower *E*_p,a_ have a lower SOMO–LUMO gap and the SOMO is localized mostly in the substituent at the α-C, while the LUMO is delocalized throughout the 1,7-dAhM system. Analysis of orbital surfaces revealed that the 1,7-dAhM system behaves mostly as two imine groups instead of an iminium–enamino polymethine system. Most importantly, the sulfur atom in Cys-Bx induces a node in C8 that jeopardizes the conjugation in the enamine function of the 2-thiazoline ring, possibly affecting its ability to stabilize and interact with radicals. Therefore, this analysis revealed that ionization resulting in SOMO and LUMO delocalized in different portions of the molecule are related to a higher antioxidant capacity and lower *E*_p,a_.

**The cysteine-betaxanthin has poor antioxidant capacity.** The characterization of Cys-Bx revealed that initial betaxanthin formation is followed by intramolecular cyclization leading to a thiazolidine ring that is oxidized in a 2e^−^,2H^+^ process to produce a 2-thiazoline ring, as inferred by mass spectrometry and ^1^H NMR spectroscopy analysis. This result has major biological implications since it is the first evidence that betaxanthins are susceptible to the nucleophilic attack of thiol nucleophiles, including the side chain of cysteine residues in peptides and proteins, which can lead to the formation of adducts. Although it is unclear how the final oxidation takes place, Cys-Bx does not scavenge ABTS^•+^ or undergo SET with the ferric-tripyridyltriazine complex. The poor antioxidant capacity of Cys-Bx and the similarity between its structure and that of Gly-Bx, except for the CH_2_S group closing the heterocyclic ring, suggests that the S atom compromises the formation of a stable radical or radical cation by oxidation of Cys-Bx. It is unlikely that Cys-Bx is formed upon use of cysteine to preserve processed beetroots and the natural occurrence of this compound is yet to be verified.

The antioxidant capacity and peak potentials of amino acid betaxanthins are not correlated with the electronic properties of their amino acid precursors. The TEAC_ABTS, 7.0_ and the TEAC_ABTS, pH 3.6_ of the betaxanthins derived from proteinogenic amino acids show modest linear correlation, as evidenced by a coefficient of determination (R^2^) of 0.80. The same is observed when comparing the TEAC_ABTS, 7.0_ with TEAC_FRAP, pH 3.6_ (R^2^ = 0.76) and the TEAC_ABTS, 3.6_ with TEAC_FRAP, 3.6_ (R^2^ = 0.82). Values of TEAC also show a poor linear correlation with the *E*_p,a_ (R^2^ < 0.5). Dwyer correlated the electronic properties of amino acid side chains, mostly inductive and field effects described by the σ_I_, σ_R_, σ_α_, and σ_F_ parameters, with their influence in the structure of proteins [90]. These parameters, derived from linear free-energy relationships, also show poor correlation with the values of TEAC or with the *E*_p,a_ of amino acid betaxanthins. Using a genetic algorithm parameterized with MKS changes of *N*1, *N*9, and all *C*OOH, HOMO and LUMO energies, the HOMO–LUMO gap, the σ_I_, σ_R_, σ_α_, and σ_F_ parameters for amino acid side chains, and *E*_p,a_, we could determine a linear model that fits the TEAC_ABTS, pH 7.0_ data reasonably well; TEAC_ABTS, 7.0_ = 2.420 − 0.004 *E*_p,a_ − 4.120 × E_LUMO_ − 4.420 MKS-*N*1, R^2^ = 0.91. Such models provide clues on which parameters, among those selected for the analysis, are important for the problem in hand. Therefore, the anodic peak potential, the energy of the LUMO, and the MKS charge of the *N*1 (which is related to the charge delocalization throughout the 1,7-dAhM system) seem to be inversely correlated with the TEAC of amino acid betaxanthins at pH 7.0.

## 5. Conclusions

The reaction of amino acids with betalamic acid produces betaxanthins whose antioxidant capacity is much higher than that of their amino acid precursors, which may represent an evolutive advantage for organisms submitted to oxidative distress. This effect is general, except for the previously unknown Cys-Bx, which is a poor antioxidant in vitro and has not been identified in vivo. The presence of ionizable side chains containing a catechol (O–H) or an indole (N–H) substituent increase the antioxidant capacity of betaxanthins by favoring their oxidation at multiple centers, which always include the 1,7-diazaheptamethinium system. Trp-Bx is an efficient antioxidant in vitro that has been found in Opuntia fruits [91,92], but its properties in vivo have not been widely investigated. The findings presented here may contribute to the development of non-phenolic semisynthetic betaxanthins for application as antioxidants and redox mediators.

## Data Availability

The data presented in this study are available in the supplementary material.

## Supplementary Materials

The following supporting information can be downloaded at: https://www.mdpi.com/article/10.3390/antiox11112259/s1, Figure S1. Chromatograms for all betaxanthins; Figure S2–S25. Mass spectra and fragmentation pattern of all betaxanthins; Figure S26. ^1^H NMR spectrum of Cys-Bx; Figure S27. ^1^H,^1^H-COSY spectrum of Cys-Bx; Figure S28. ^1^H NMR spectrum of Lys-Bx; Figure S29. ^1^H NMR spectrum of ε-Lys-Bx; Figure S30. Raw data FRAP assay. Figures S31–S33. Raw data ABTS assay; Figure S34. Cyclic voltammograms of betaxanthins; Figure S35. Background-corrected square wave voltammetry of Pro-Bx; Table S1. Electrochemical properties of betaxanthins; Table S2. Estimated p*K*_a_ values of betaxanthins.
