# Supplementary material for: Structural Effects on the Antioxidant Properties of Amino Acid Betaxanthins
Source: Antioxidants (Basel). 2022 Nov 16;11(11):2259. doi: 10.3390/antiox11112259 (PMC9686915; doi:10.3390/antiox11112259)
Supplement: Supplementary file 1 [file antioxidants-11-02259-s001.zip › antioxidants-2031267-supplementary.pdf]

SUPPLEMENTARY MATERIAL

Structural Effects on the Antioxidant Properties of Amino Acid Betaxanthins

Larissa C. Esteves, Caroline O. Machado, Letícia C. P. Gonçalves, Victor F. Cavalcante, Guilherme Obeid, Thiago C. Correra, and Erick L. Bastos

Summary

1. Chromatograms S2
2. ESI-qTOF-MS/MS spectra and fragmentation pattern S3
3. NMR spectra S27
4. Ferric-reducing antioxidant power (FRAP) S31
5. Antiradical capacity, TEAC/ABTS$^{•+}$ assay S32
6. Antioxidant capacity of Cys-Bx S34
7. Cyclic voltammograms S35

1. Chromatograms

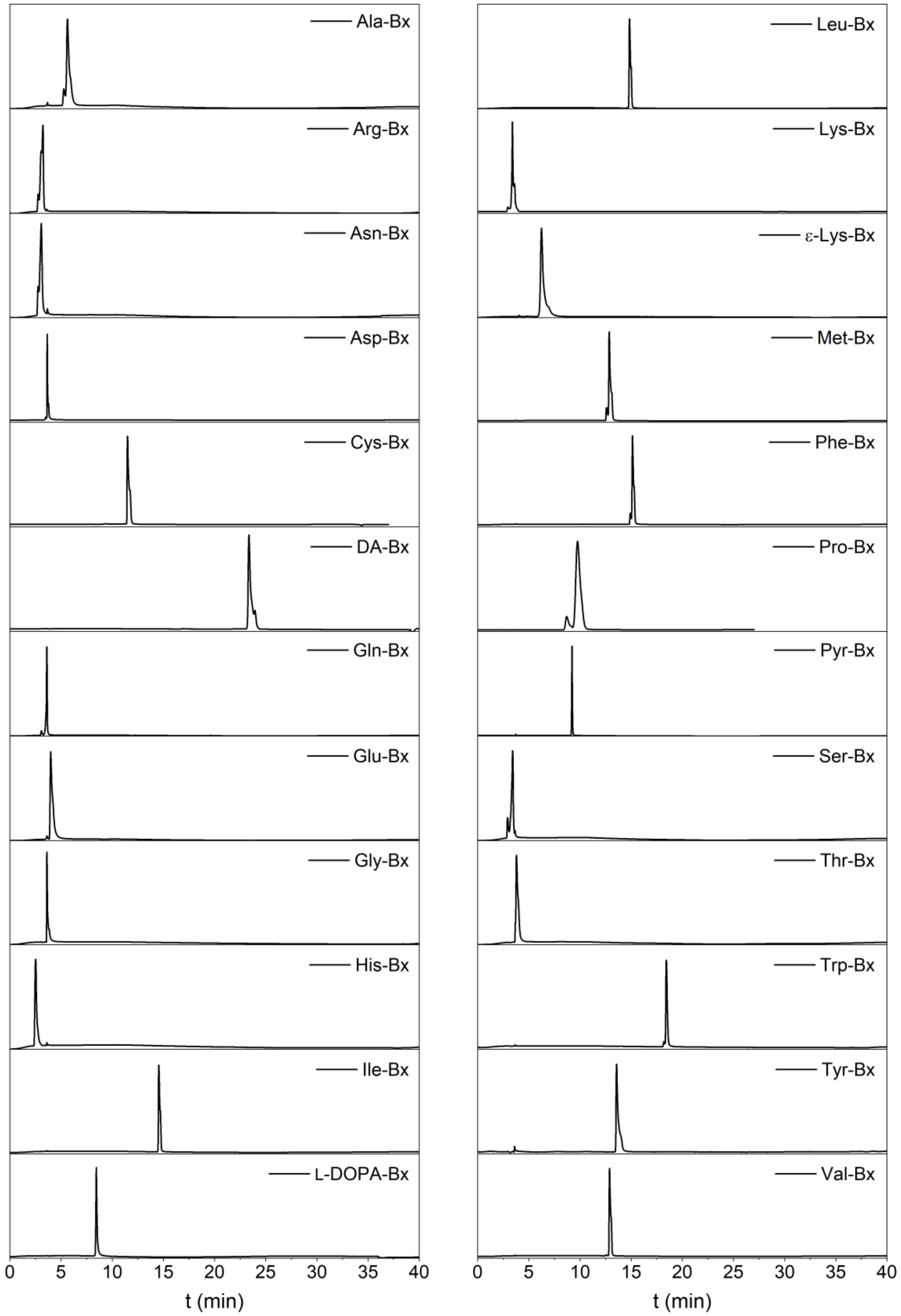

Figure S1. Chromatograms obtained with UV-Vis absorption detection set at 480 nm for all betaxanthins, except Cys-Bx that was monitored at 495 nm.

2. ESI-qTOF-MS/MS spectra and fragmentation pattern

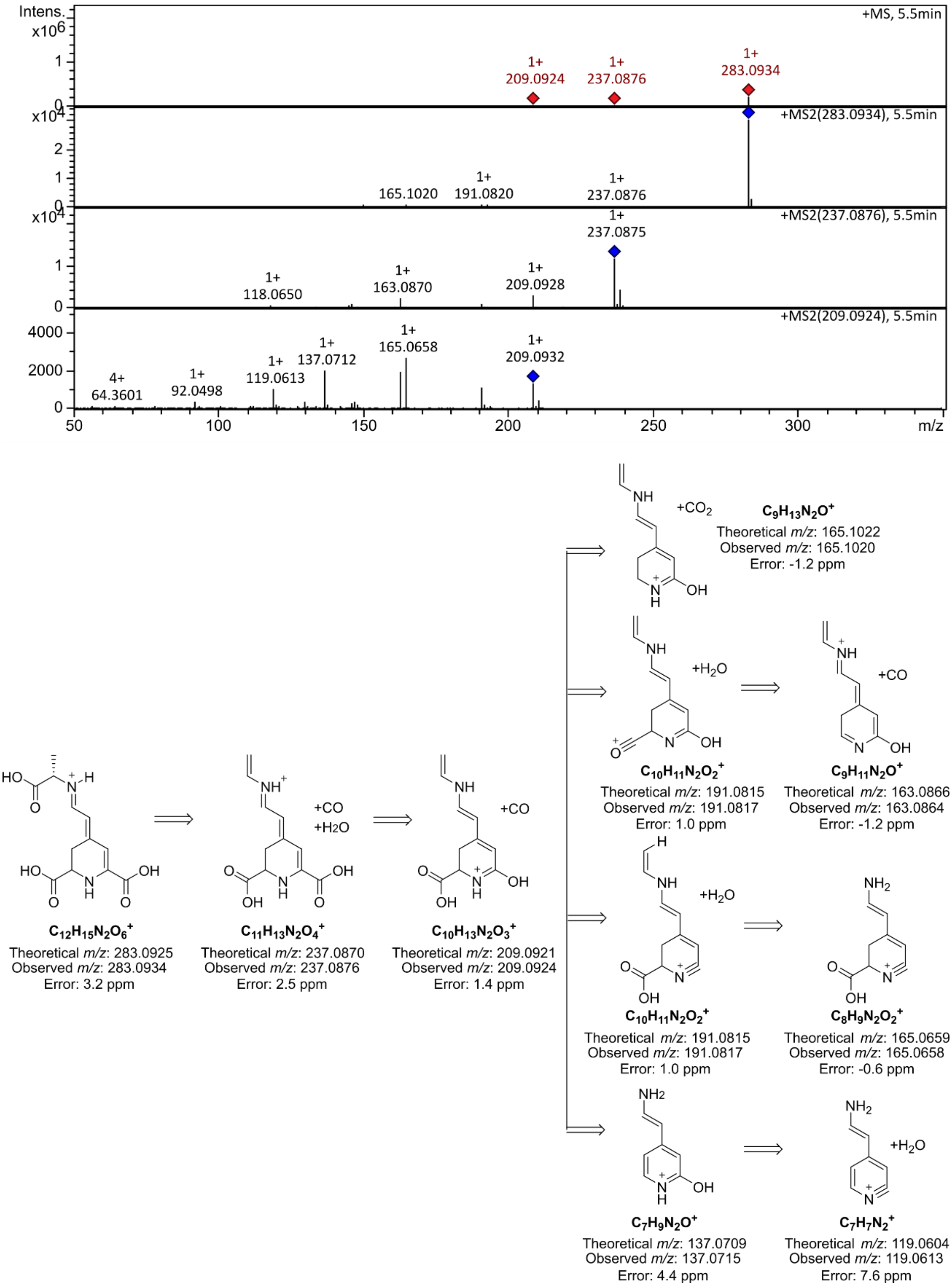

Figure S2. ESI-qTOF-MS/MS spectra and fragmentation pattern of L-alanine betaxanthin (Ala-Bx).

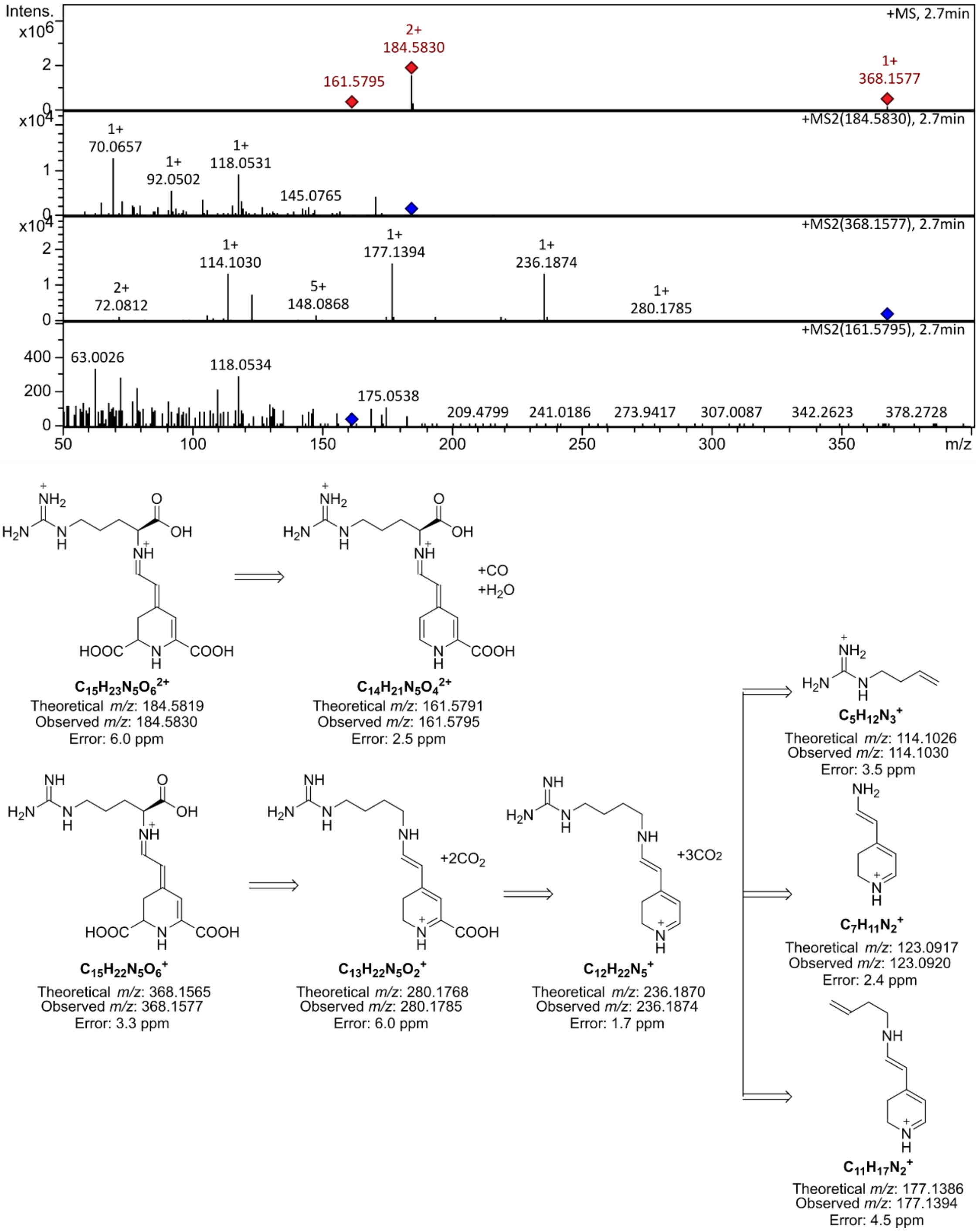

Figure S3. ESI-qTOF-MS/MS spectra and fragmentation pattern of L-arginine-betaxanthin (Arg-Bx)

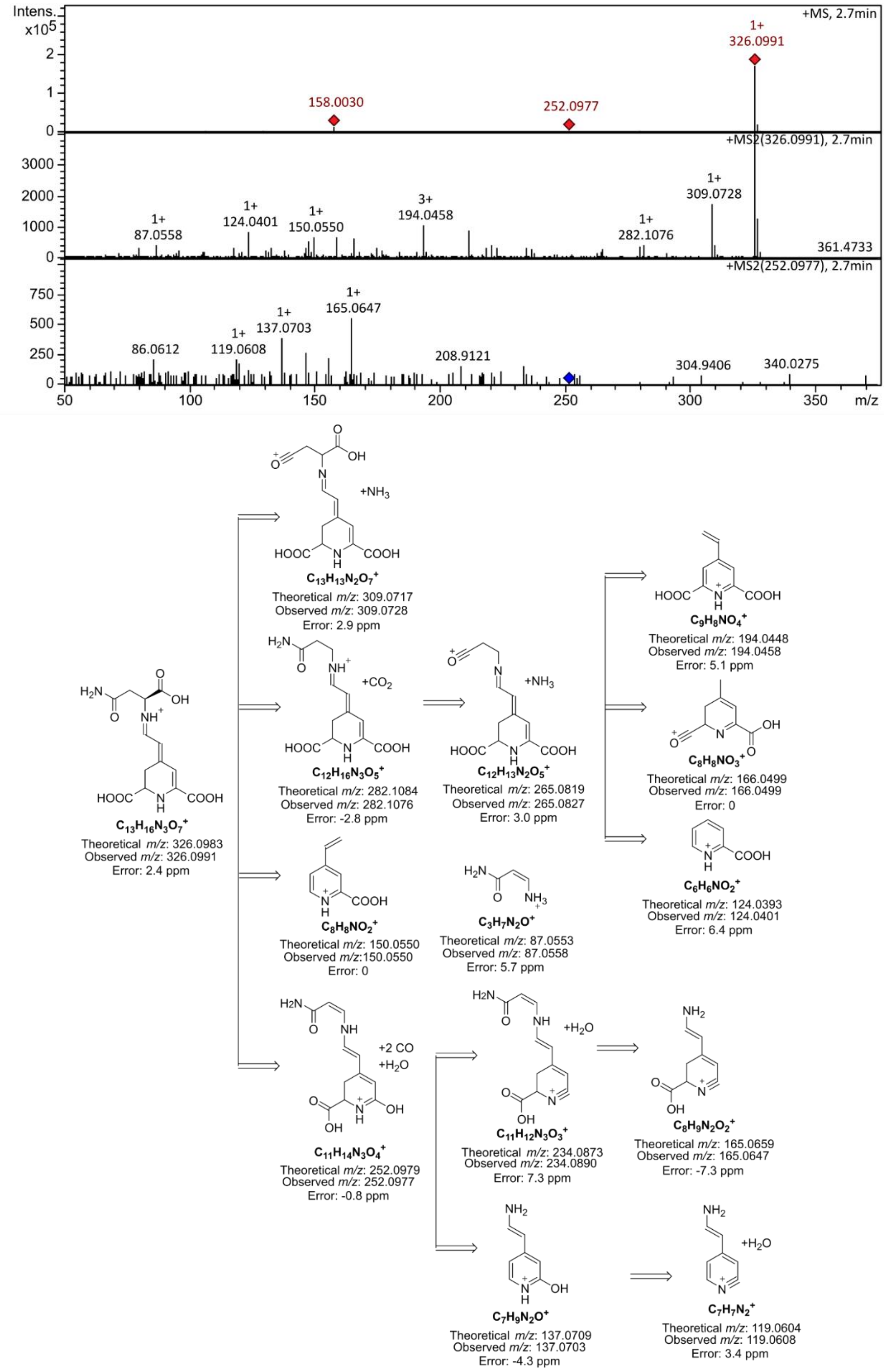

Figure S4. ESI-qTOF-MS/MS spectra and fragmentation pattern of L-asparagine-betaxanthin (Asn-Bx).

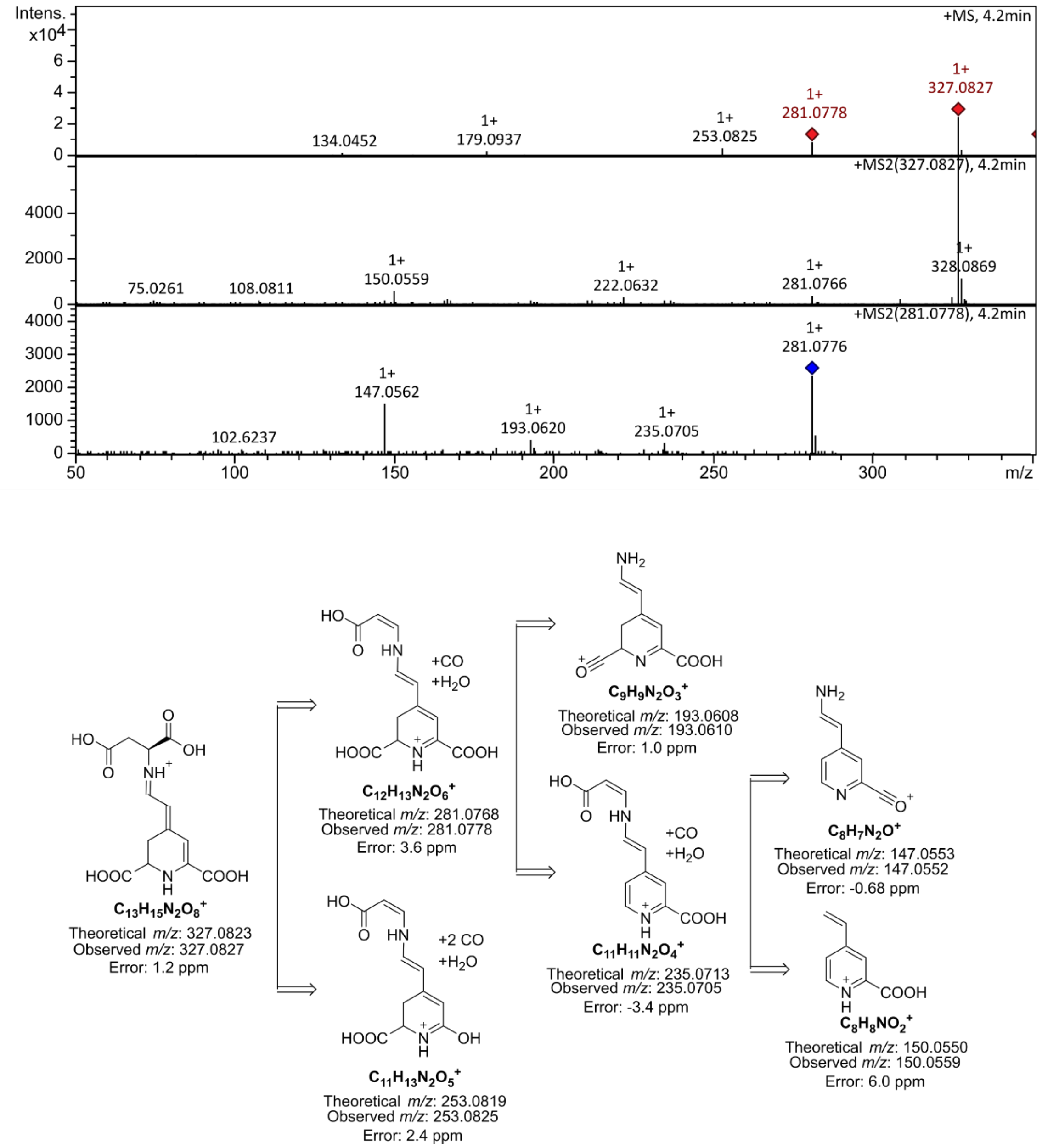

Figure S5. ESI-qTOF-MS/MS spectra and fragmentation pattern of L-aspartic acid-betaxanthin (Asp-Bx).

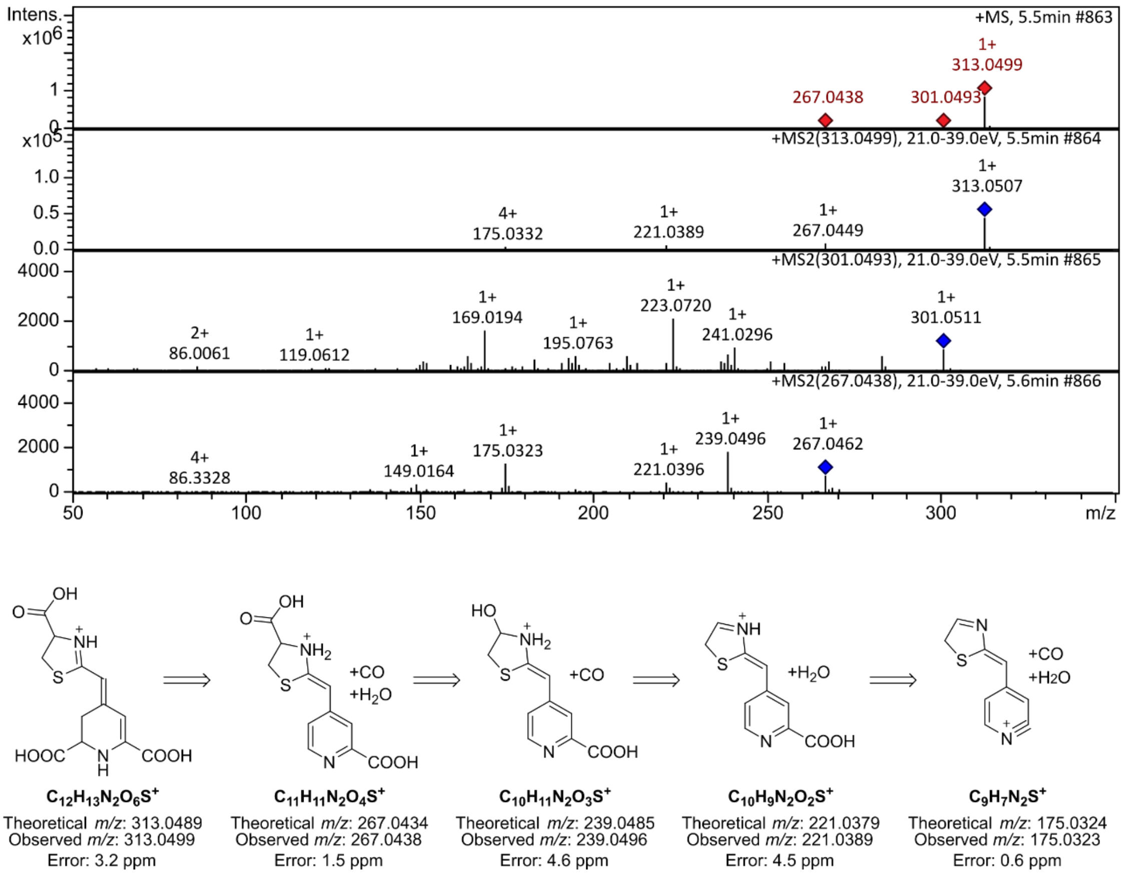

Figure S6. ESI-qTOF-MS/MS spectra and fragmentation pattern of L-cysteine-betaxanthin (Cys-Bx).

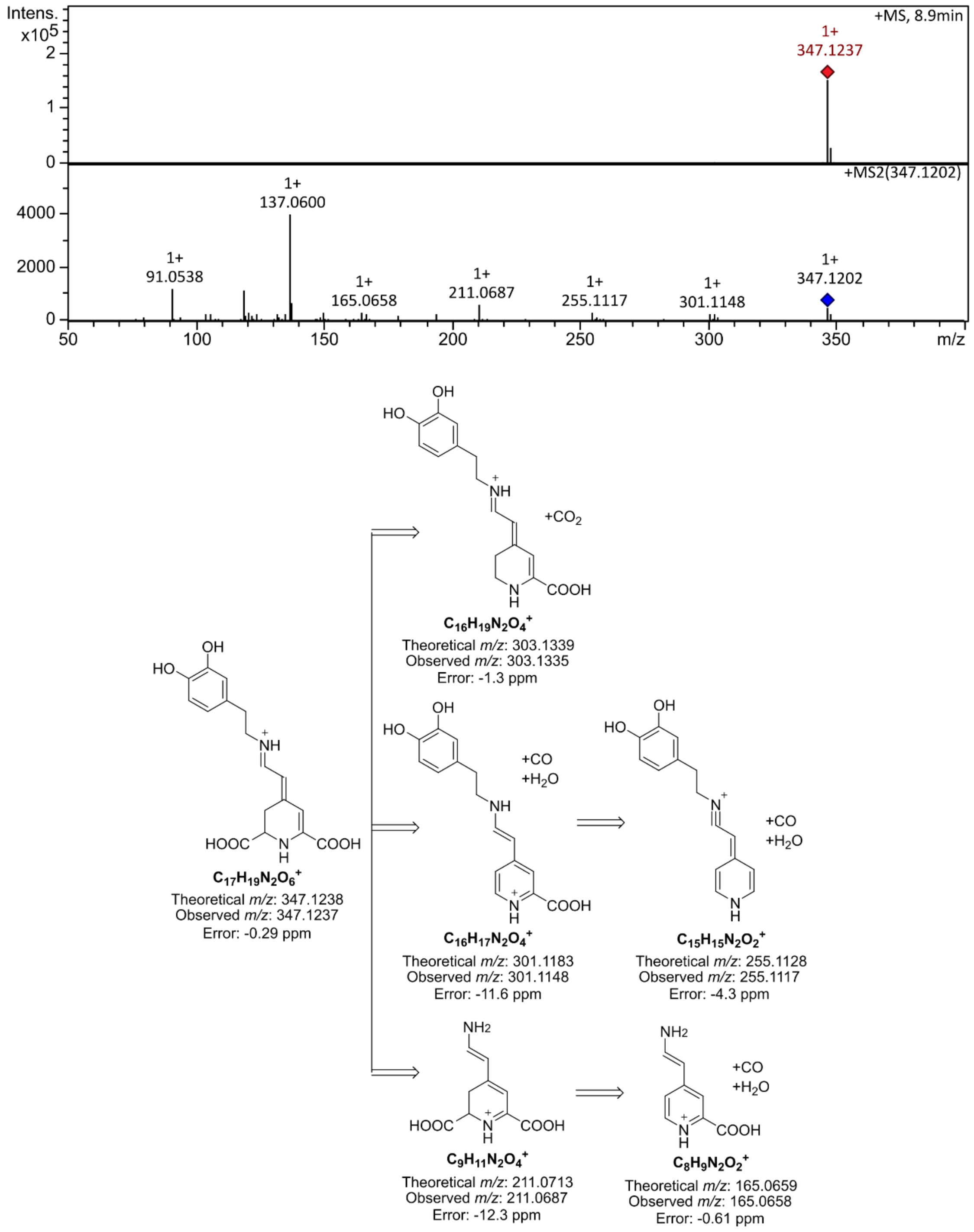

Figure S7. ESI-qTOF-MS/MS spectra and fragmentation pattern of L-dopamine-betaxanthin (DA-Bx).

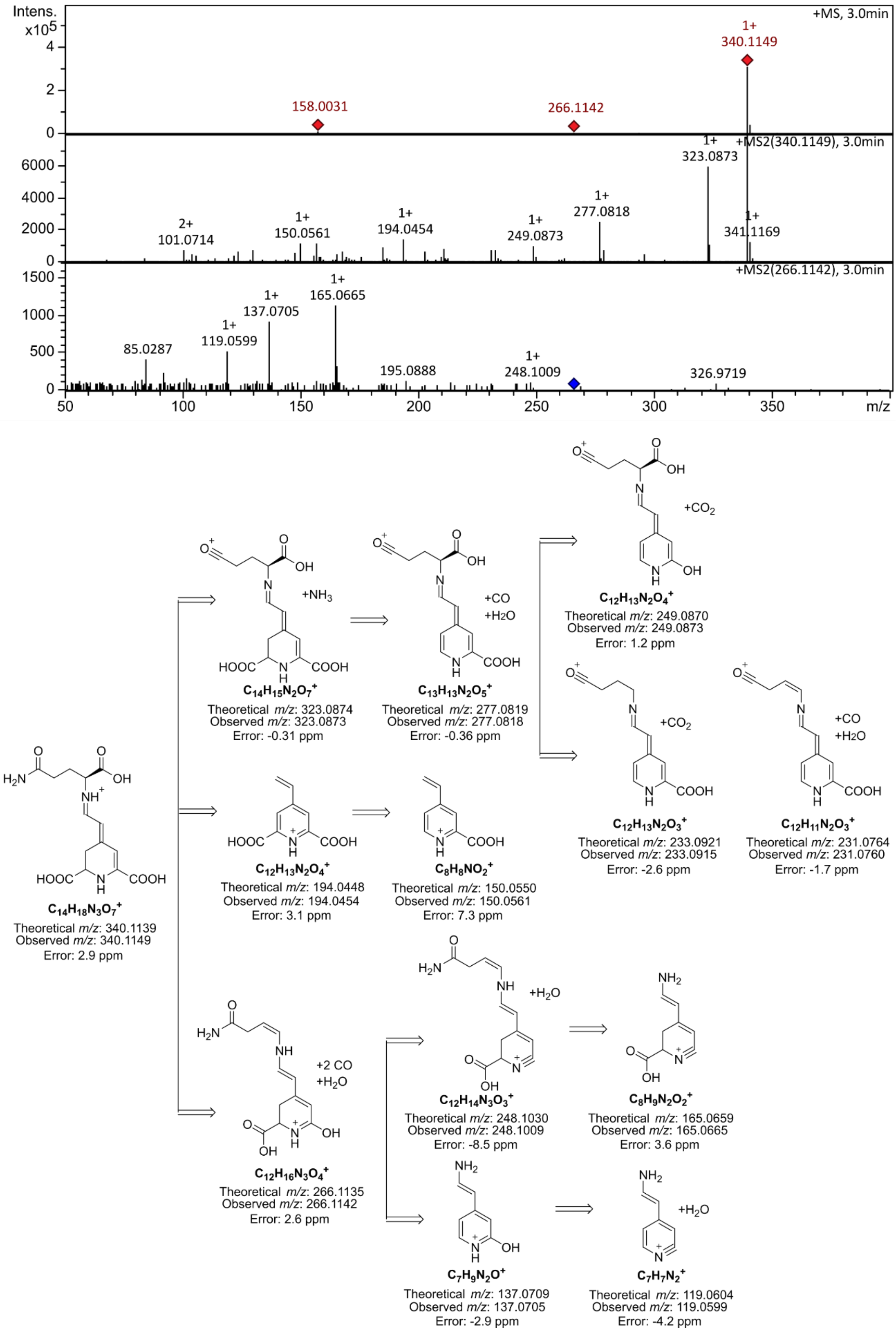

Figure S8. ESI-qTOF-MS/MS spectra and fragmentation pattern of L-glutamine-betaxanthin (Gln-Bx).

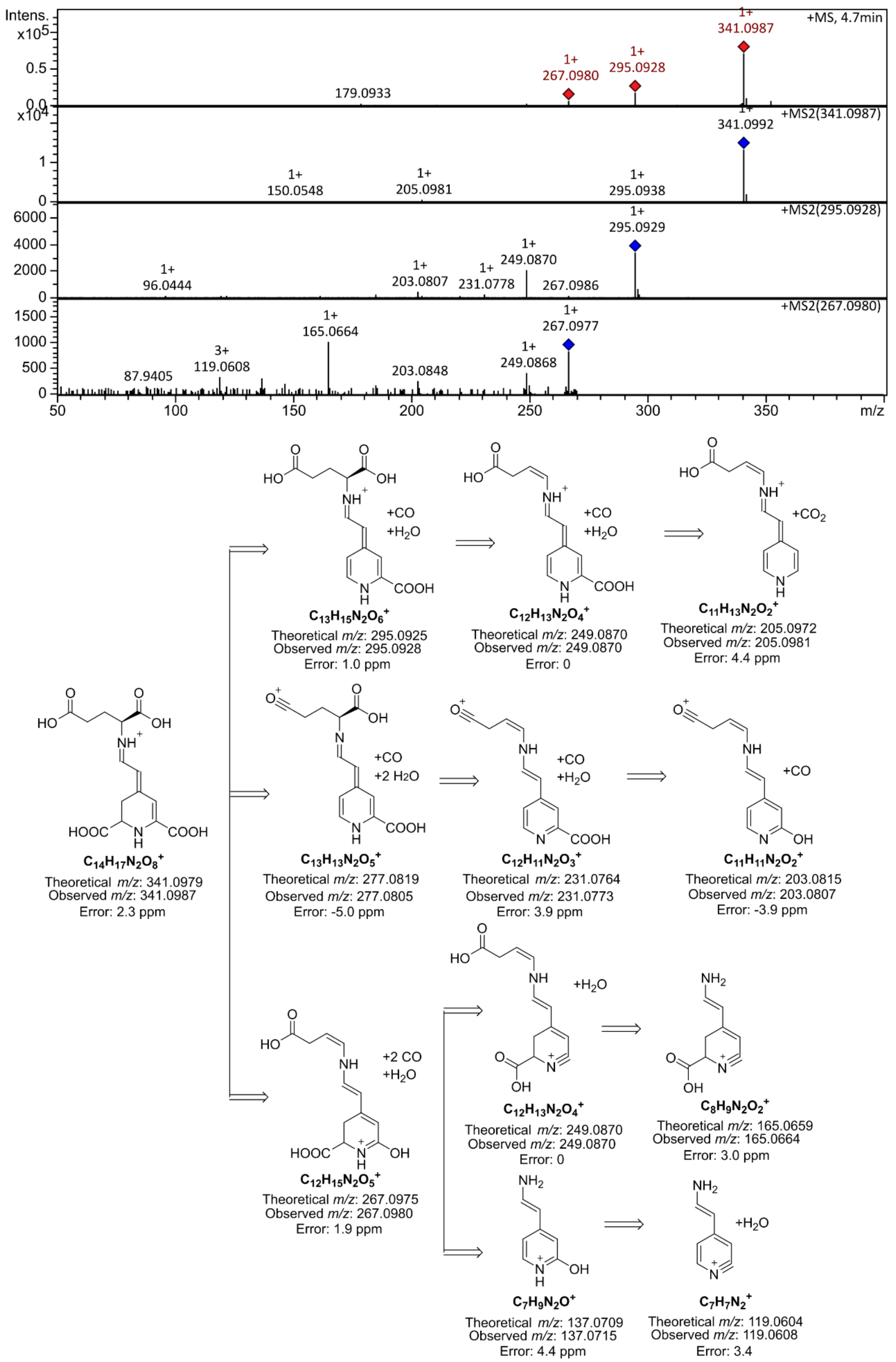

Figure S9. ESI-qTOF-MS/MS spectra and fragmentation pattern of L-glutamic acid-betaxanthin (Glu-Bx).

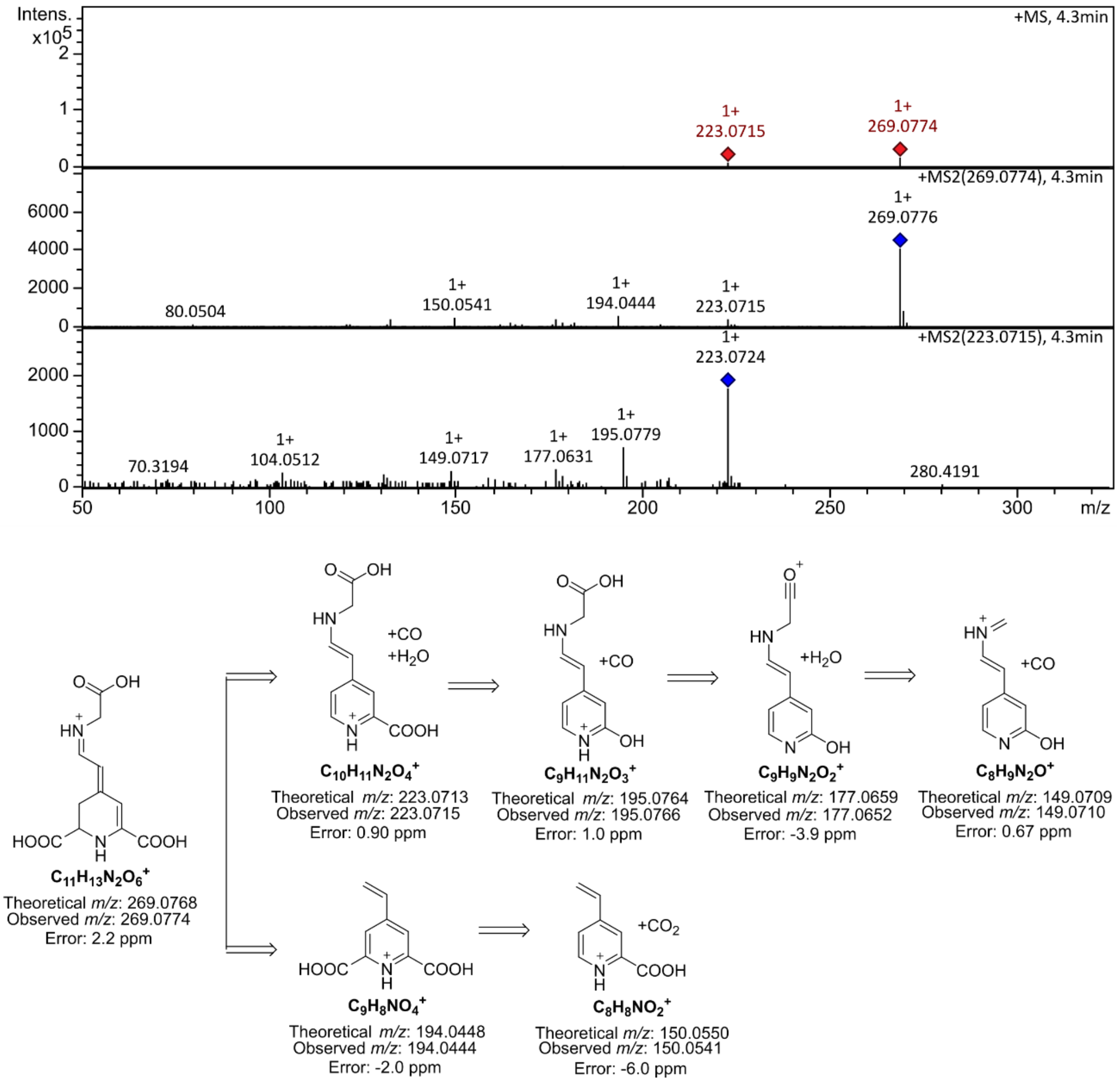

Figure S10. ESI-qTOF-MS/MS spectra and fragmentation pattern of L-glycine-betaxanthin (Gly-Bx).

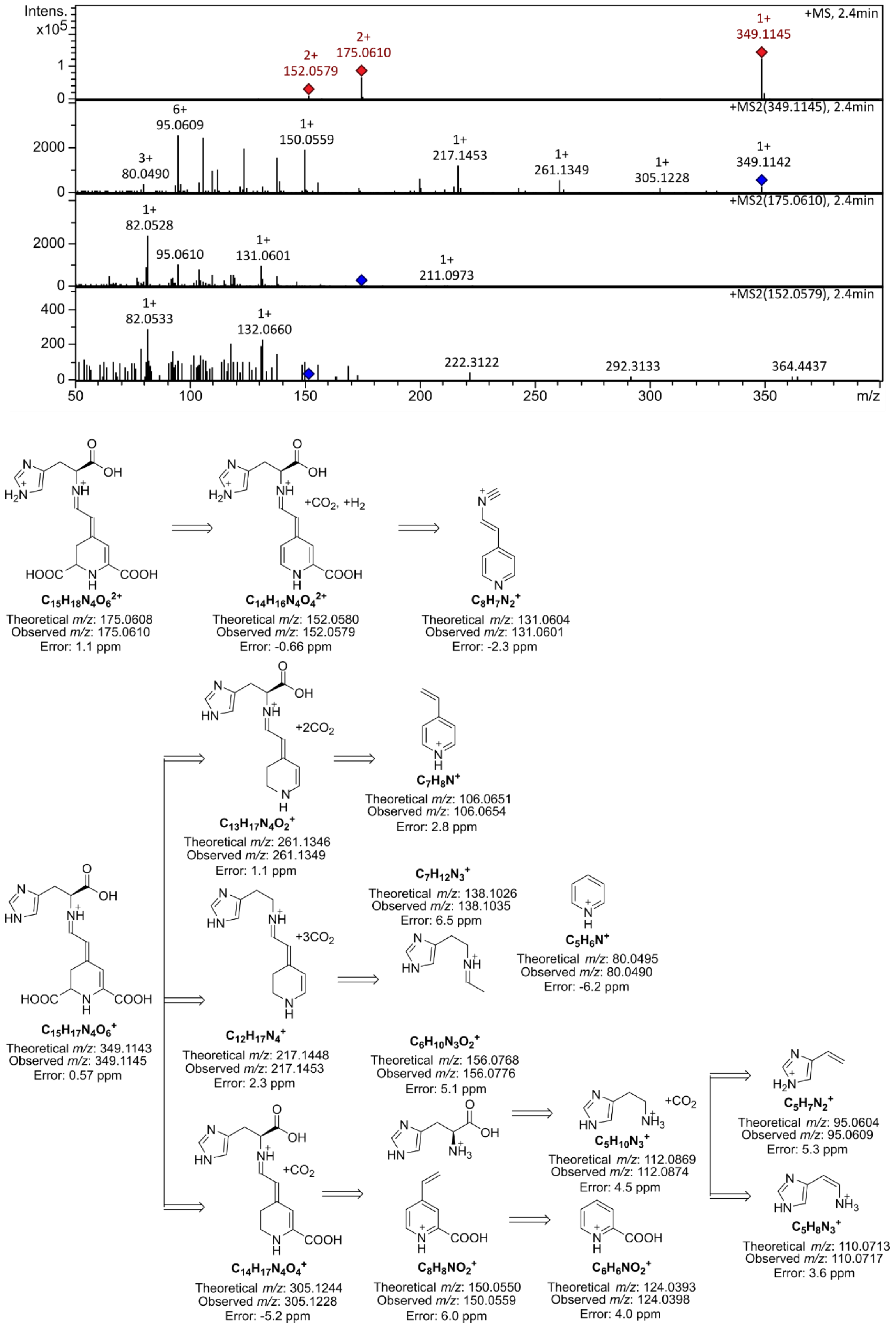

Figure S11. ESI-qTOF-MS/MS spectra and fragmentation pattern of L-histidine-betaxanthin (His-Bx).

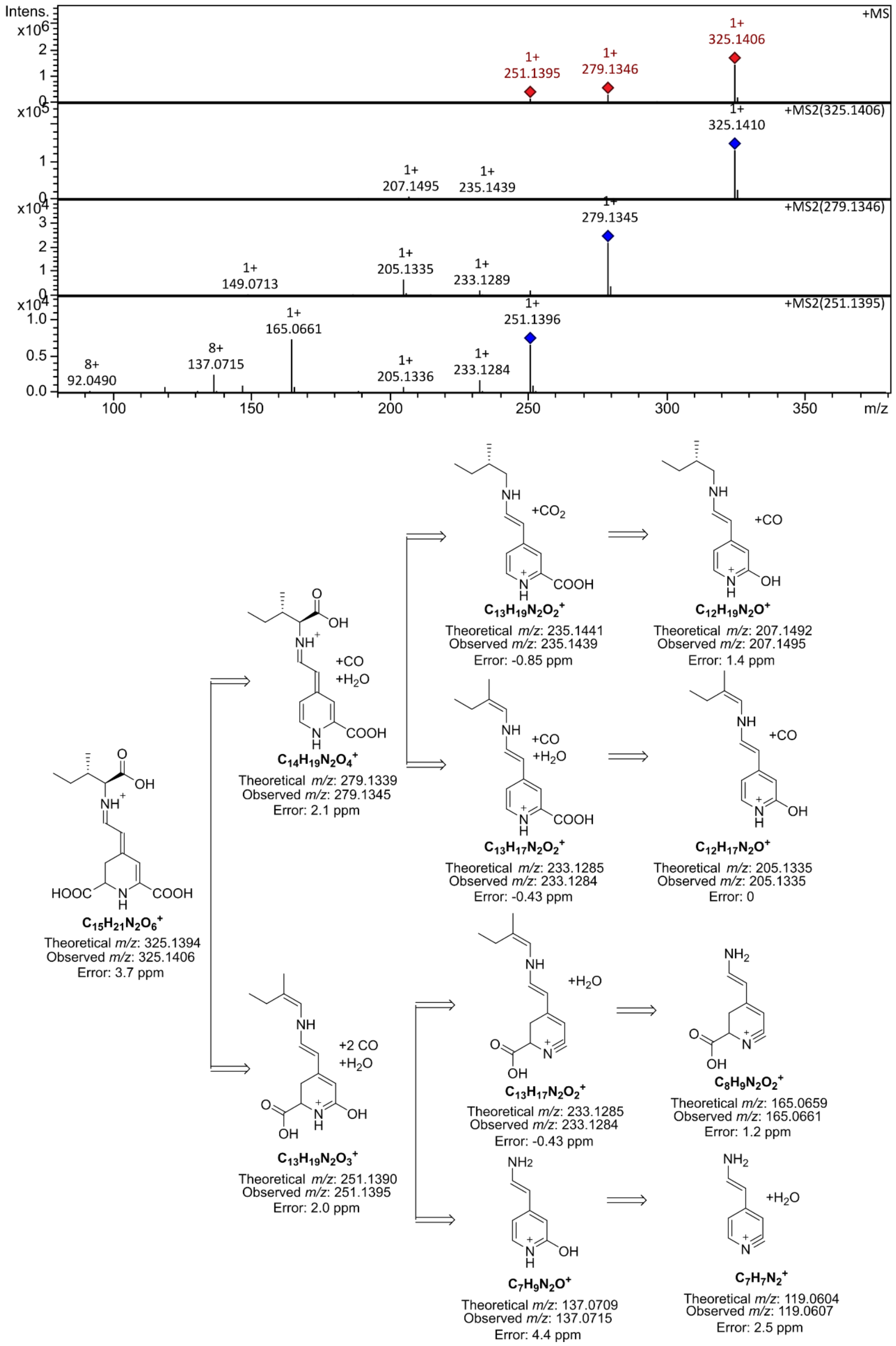

Figure S12. ESI-qTOF-MS/MS spectra and fragmentation pattern of L-isoleucine-betaxanthin (Ile-Bx).

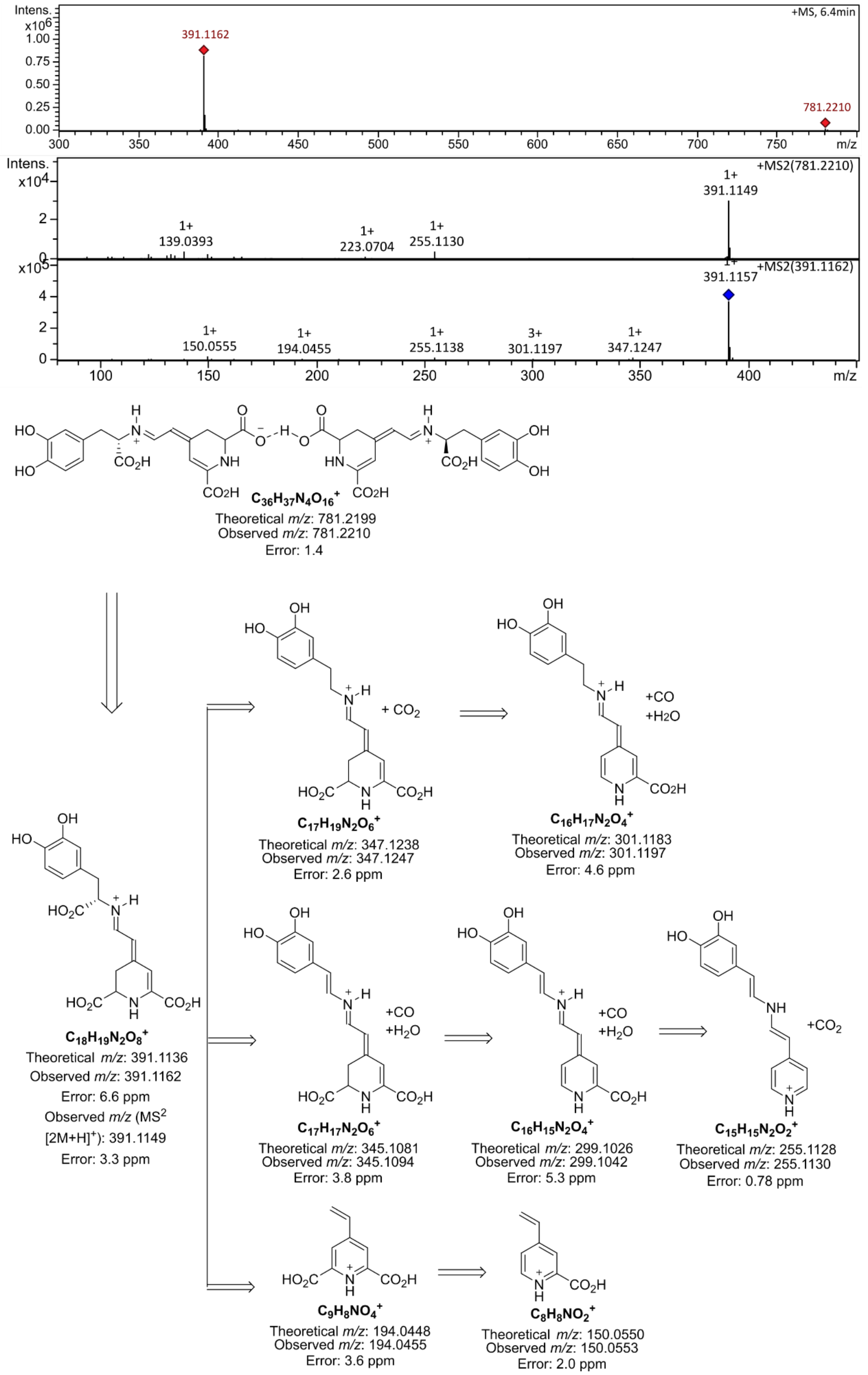

Figure S13. ESI-qTOF-MS/MS spectra and fragmentation pattern of L-DOPA-betaxanthin (L-DOPA-Bx).

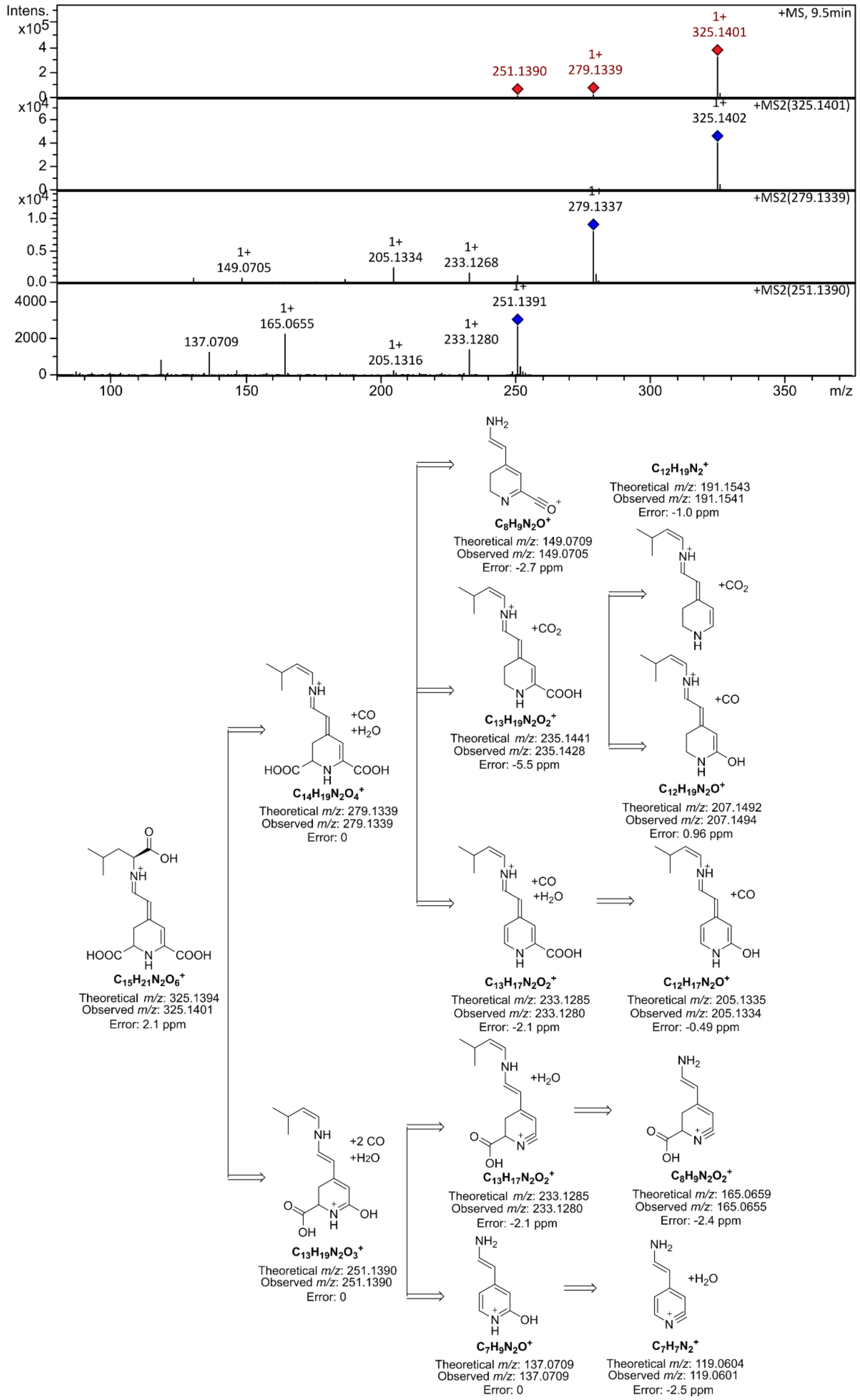

Figure S14. ESI-qTOF-MS/MS spectra and fragmentation pattern of L-leucine-betaxanthin (Leu-Bx).

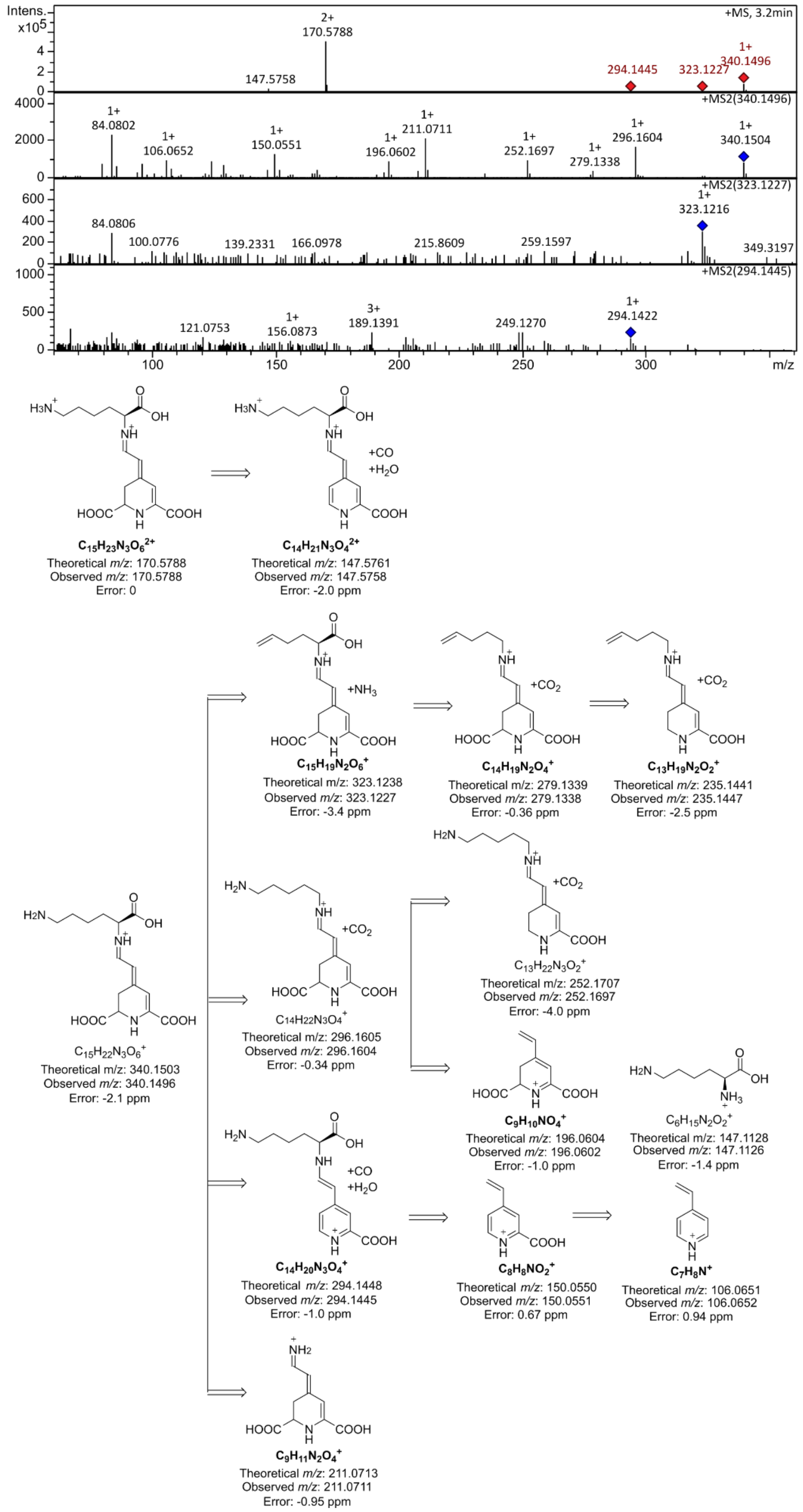

Figure S15. ESI-qTOF-MS/MS spectra and fragmentation pattern of L-lysine-betaxanthin (Lys-Bx).

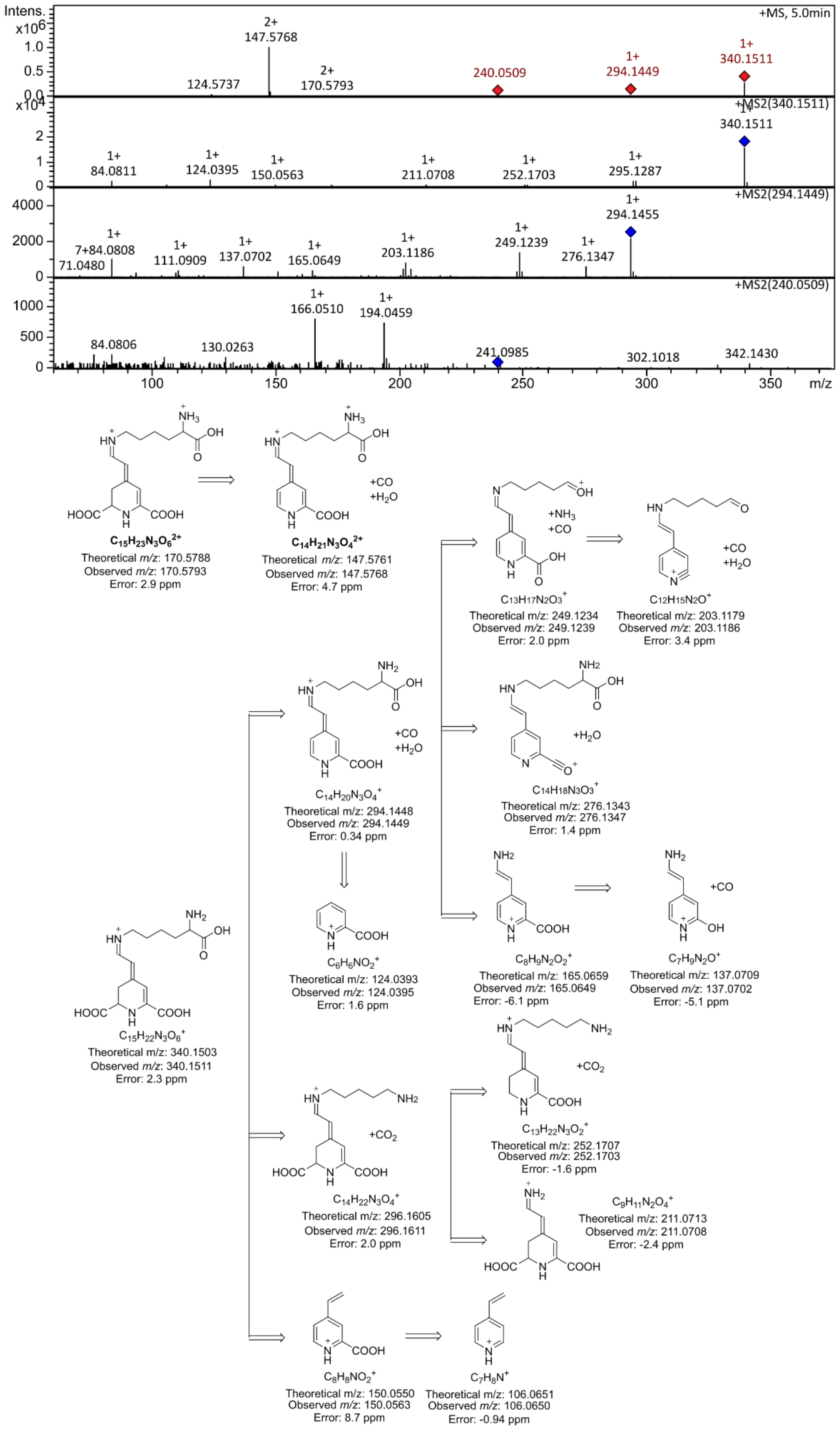

Figure S16. ESI-qTOF-MS/MS spectra and fragmentation pattern of L-ε-Lysine-betaxanthin (ε-Lys-Bx).

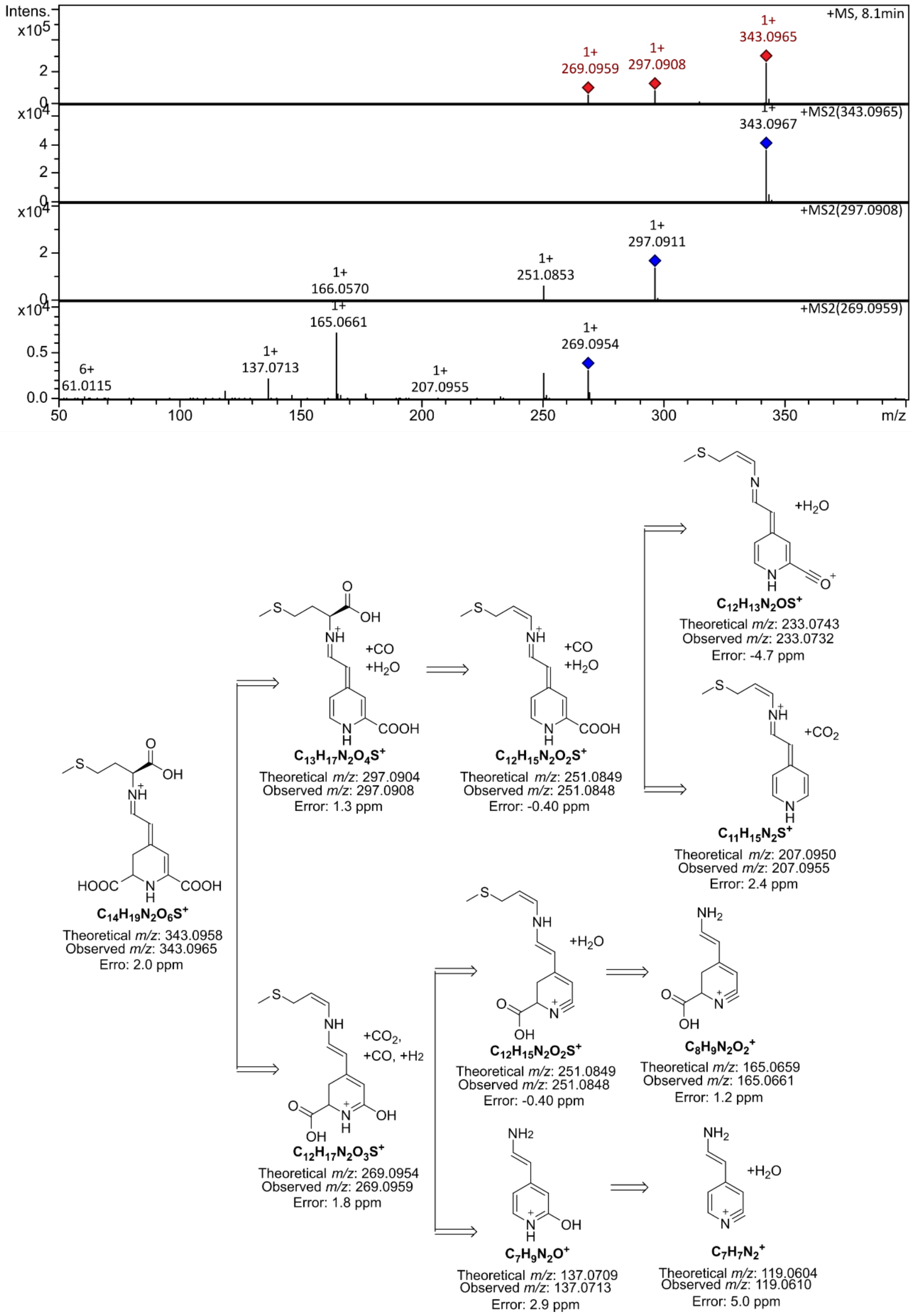

Figure S17. ESI-qTOF-MS/MS spectra and fragmentation pattern of L-methionine-betaxanthin (Met-Bx).

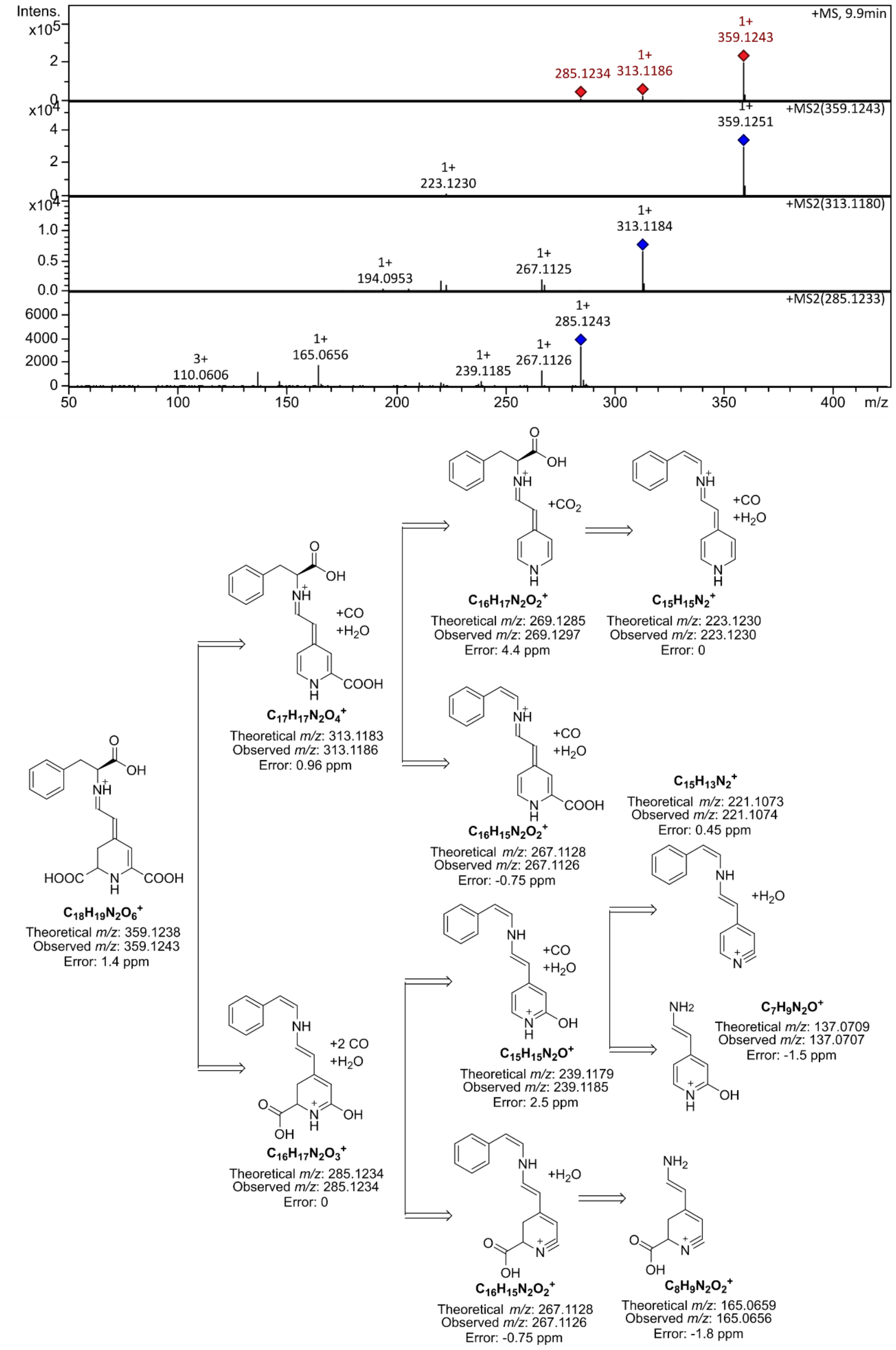

Figure S18. ESI-qTOF-MS/MS spectra and fragmentation pattern of L-phenylalanine-betaxanthin (Phe-Bx).

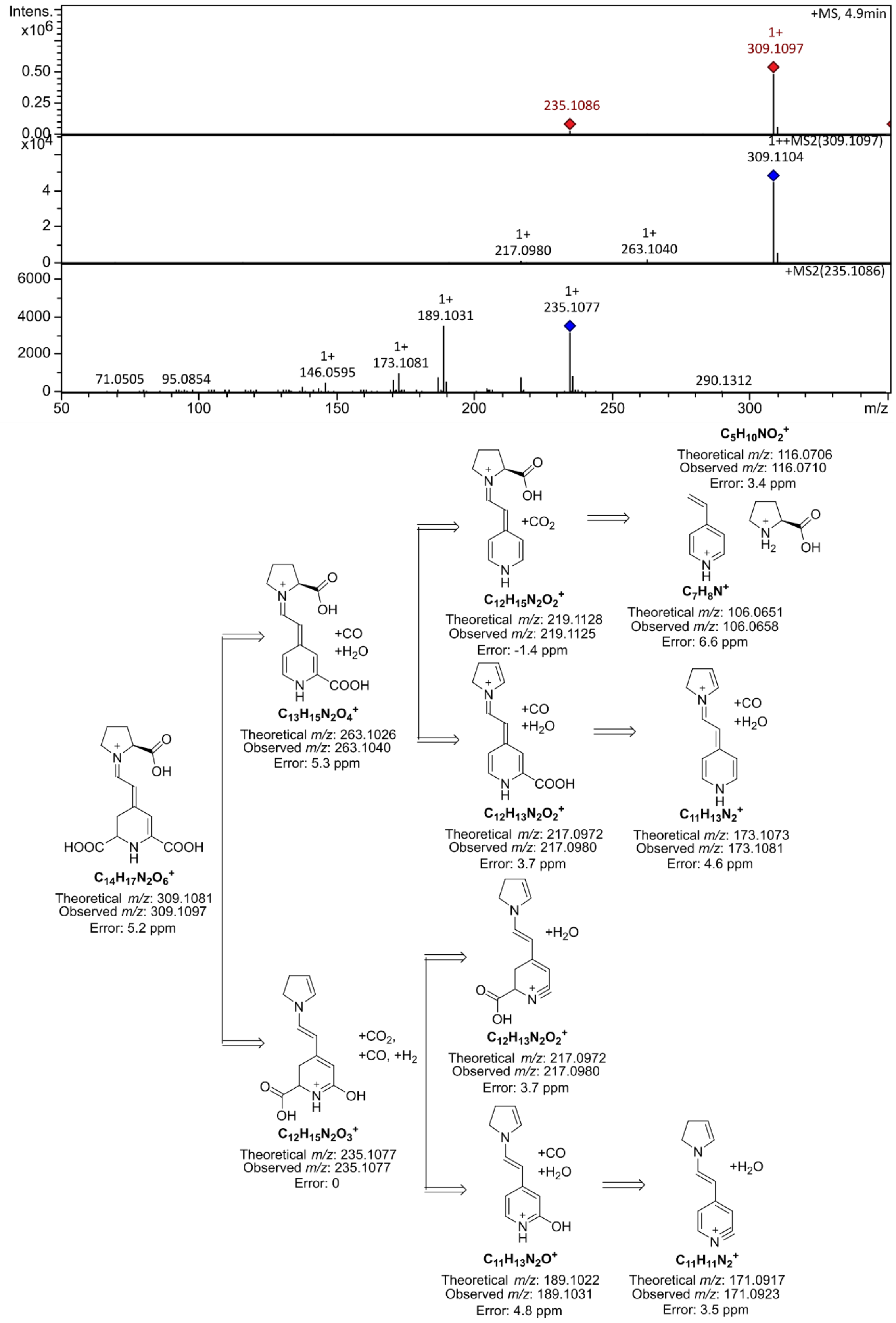

Figure S19. ESI-qTOF-MS/MS spectra and fragmentation pattern of L-proline-betaxanthin (Pro-Bx).

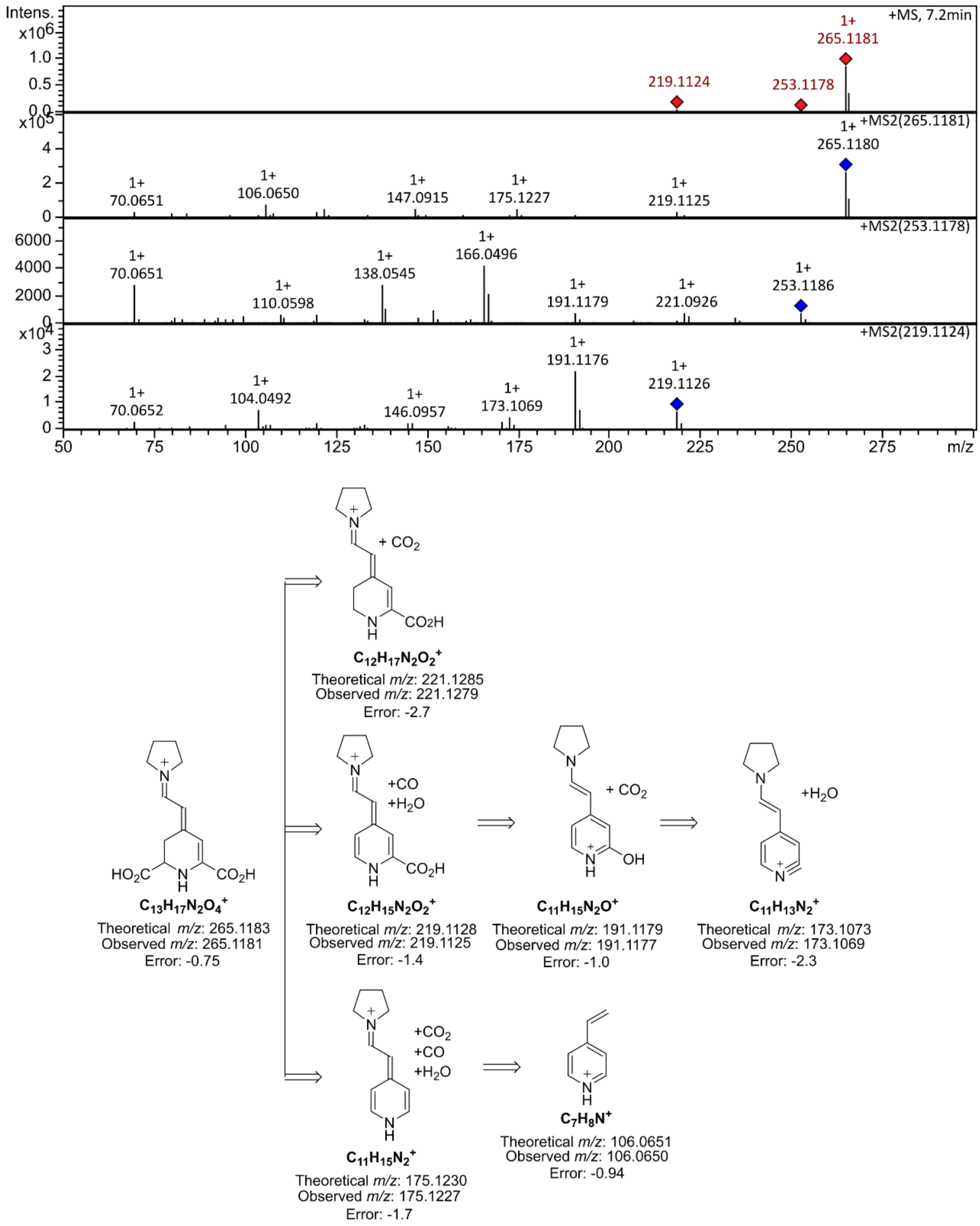

Figure S20. ESI-qTOF-MS/MS spectra and fragmentation pattern of pyrrolidine-betaxanthin (Pyr-Bx).

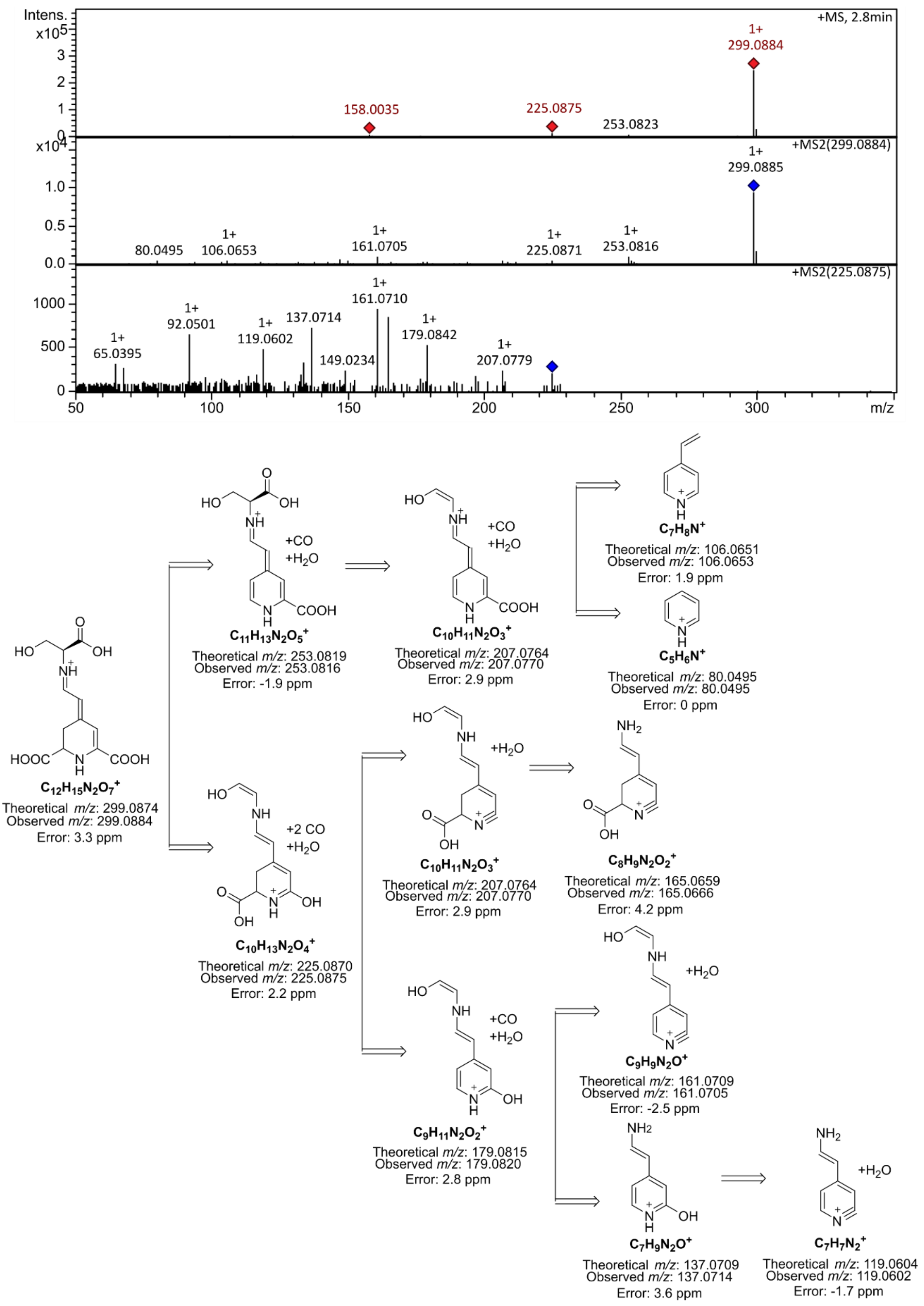

Figure S21. ESI-qTOF-MS/MS spectra and fragmentation pattern of L-serine-betaxanthin (Ser-Bx).

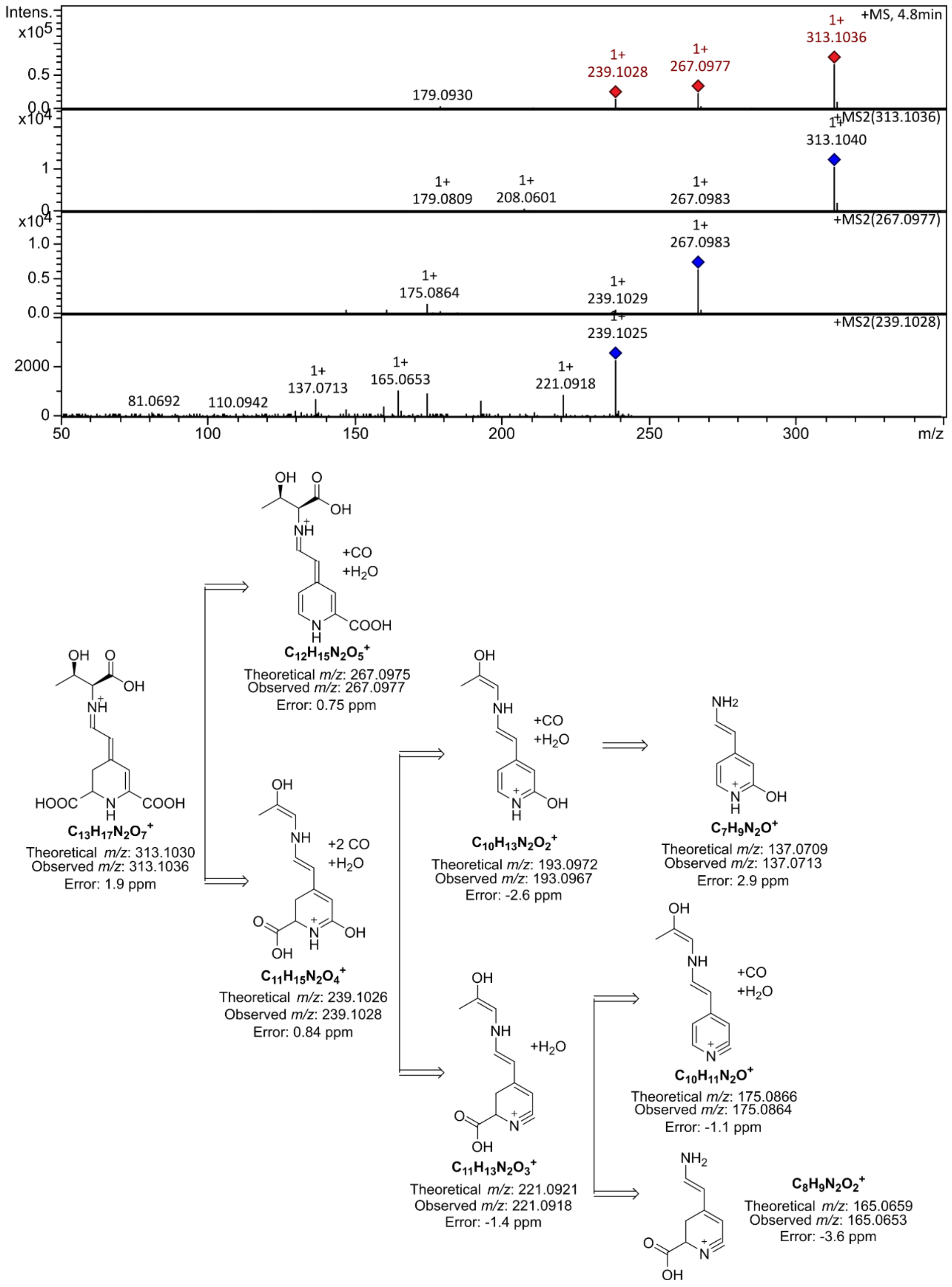

Figure S22. ESI-qTOF-MS/MS spectra and fragmentation pattern of L-threonine-betaxanthin (Thr-Bx).

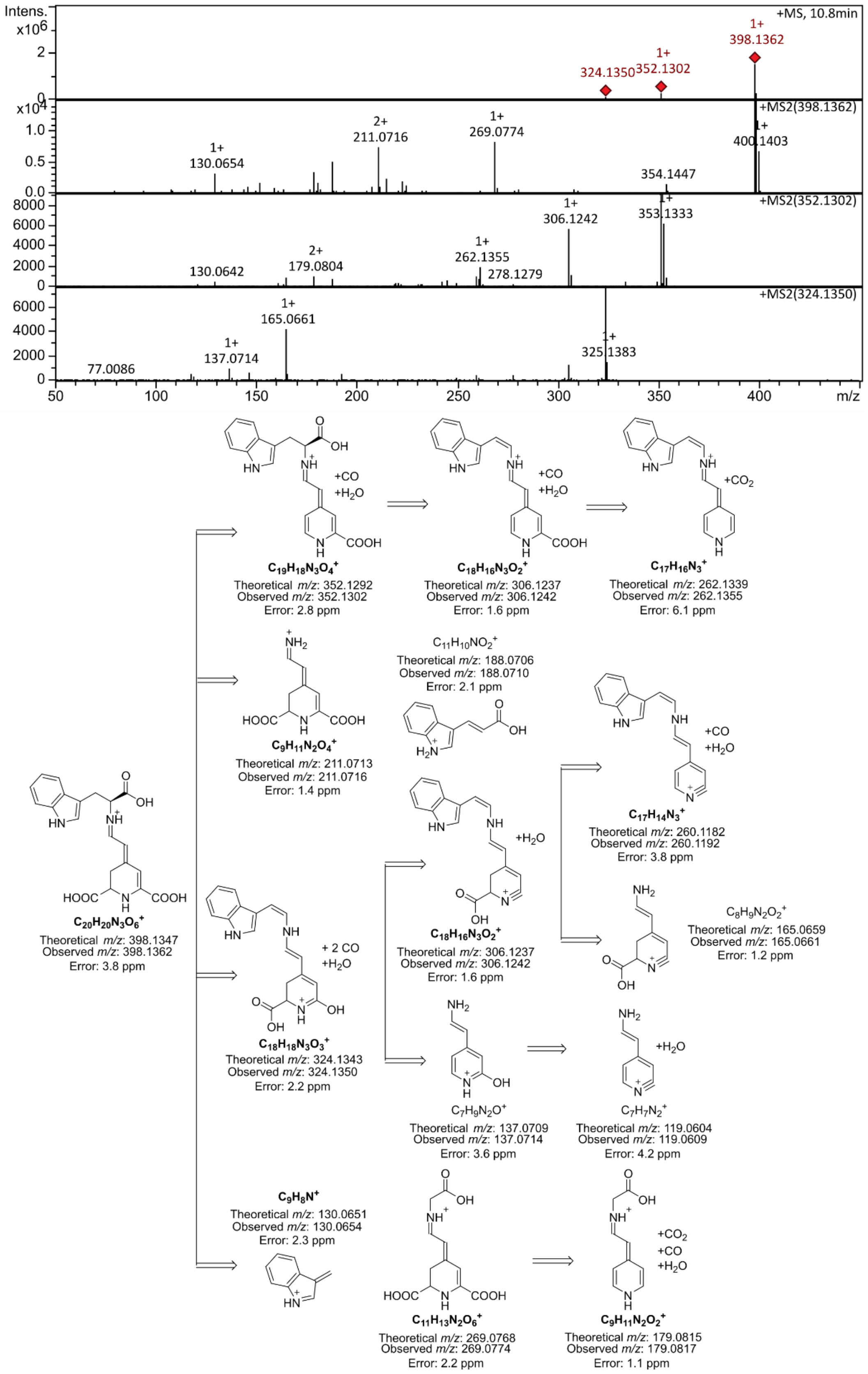

Figure S23. ESI-qTOF-MS/MS spectra and fragmentation pattern of L-tryptophan-betaxanthin (Trp-Bx).

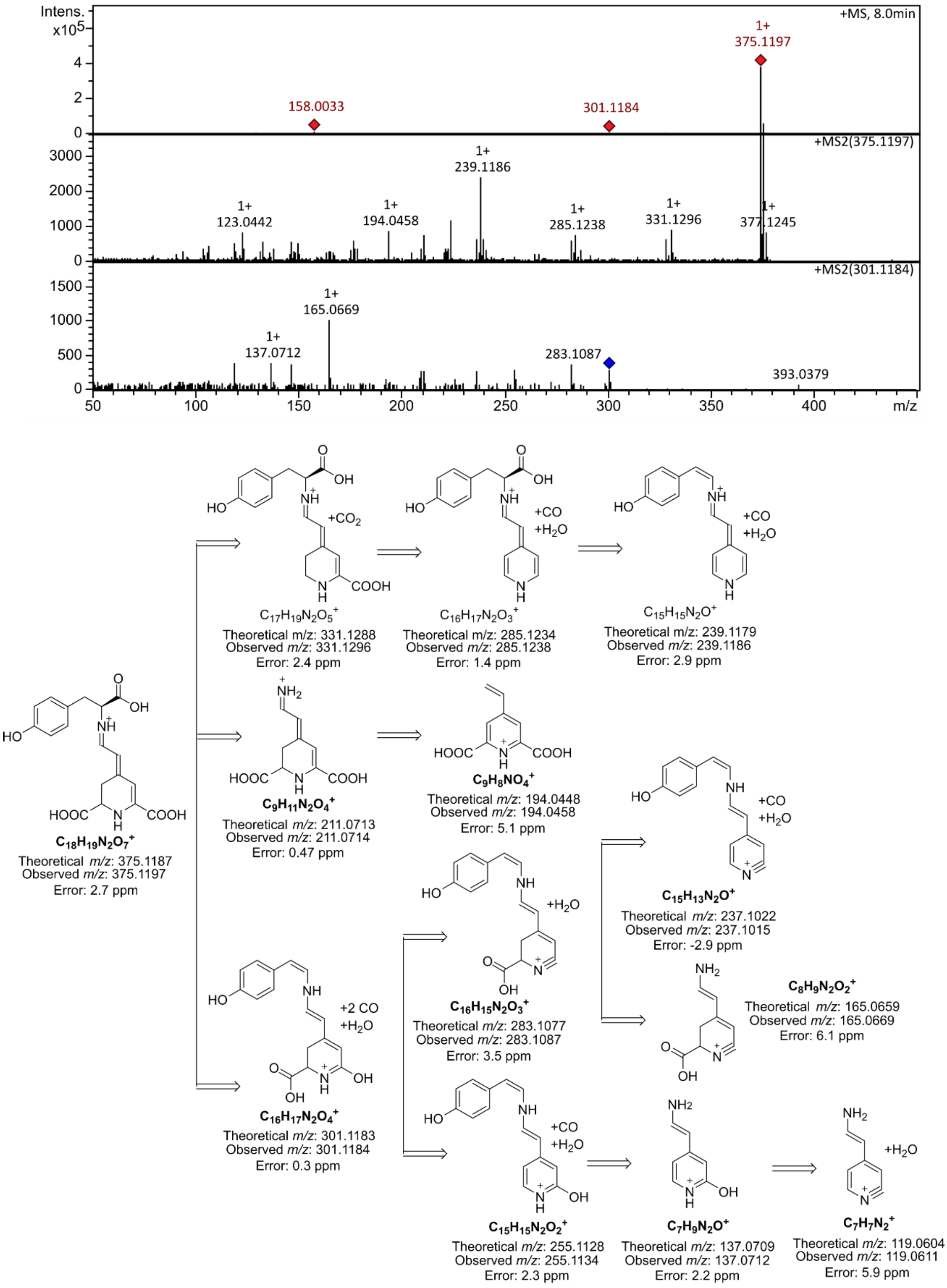

Figure S24. ESI-qTOF-MS/MS spectra and fragmentation pattern of L-tyrosine-betaxanthin (Tyr-Bx).

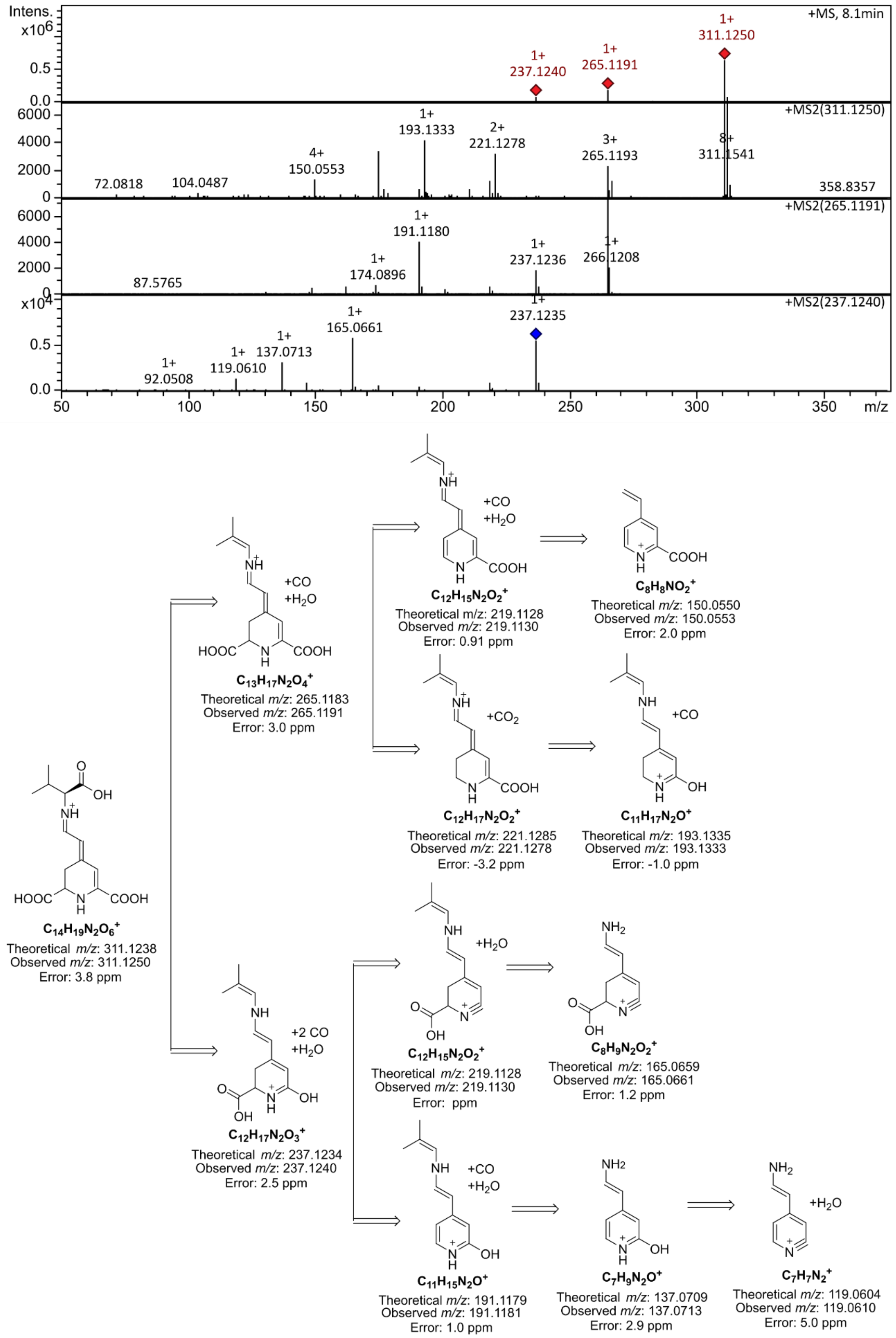

Figure S25. ESI-qTOF-MS/MS spectra and fragmentation pattern of L-valine-betaxanthin (Val-Bx).

3. NMR spectra

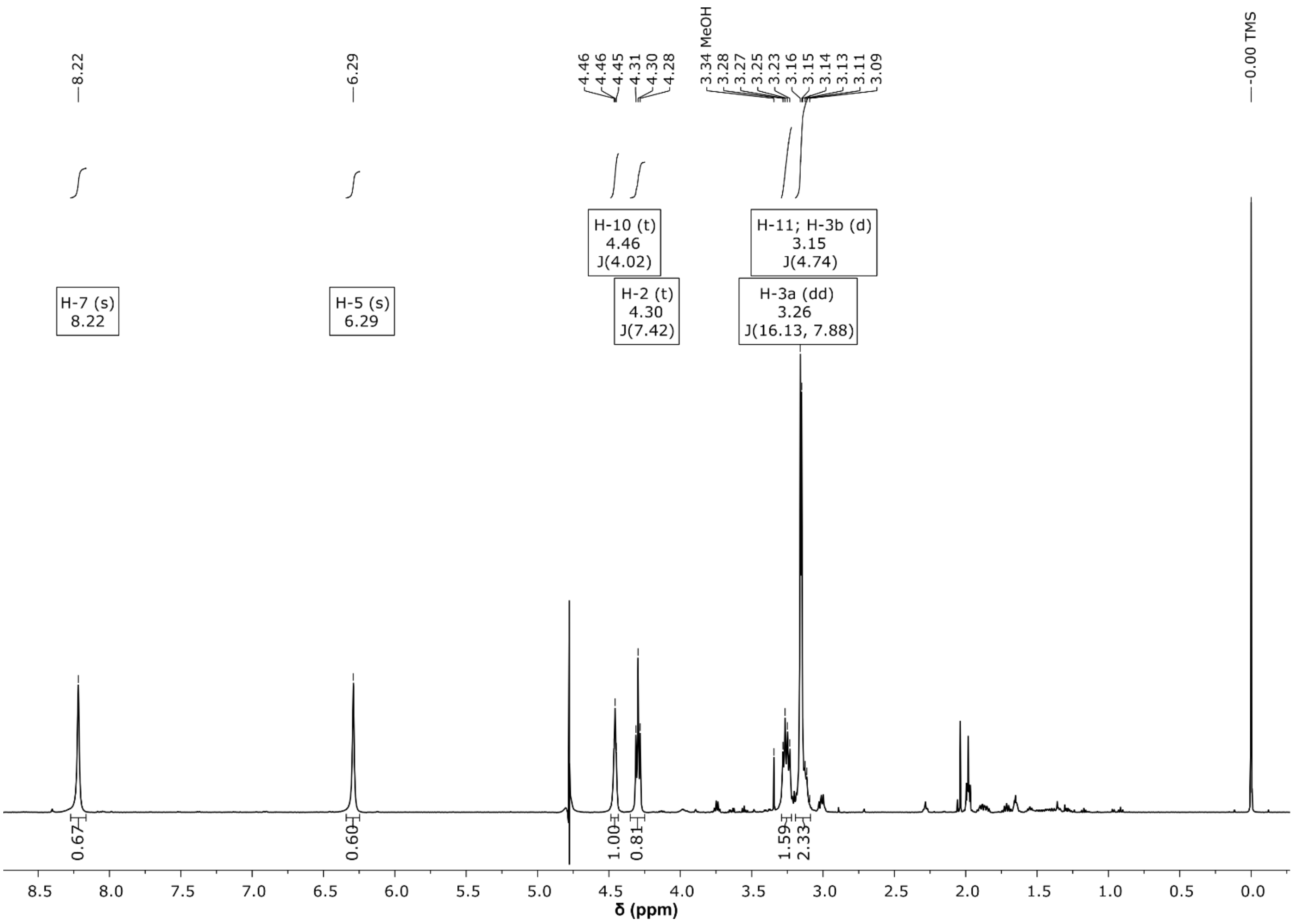

Figure S26. ^{1}H NMR spectrum of Cys-Bx, in D_2O at 25 °C.

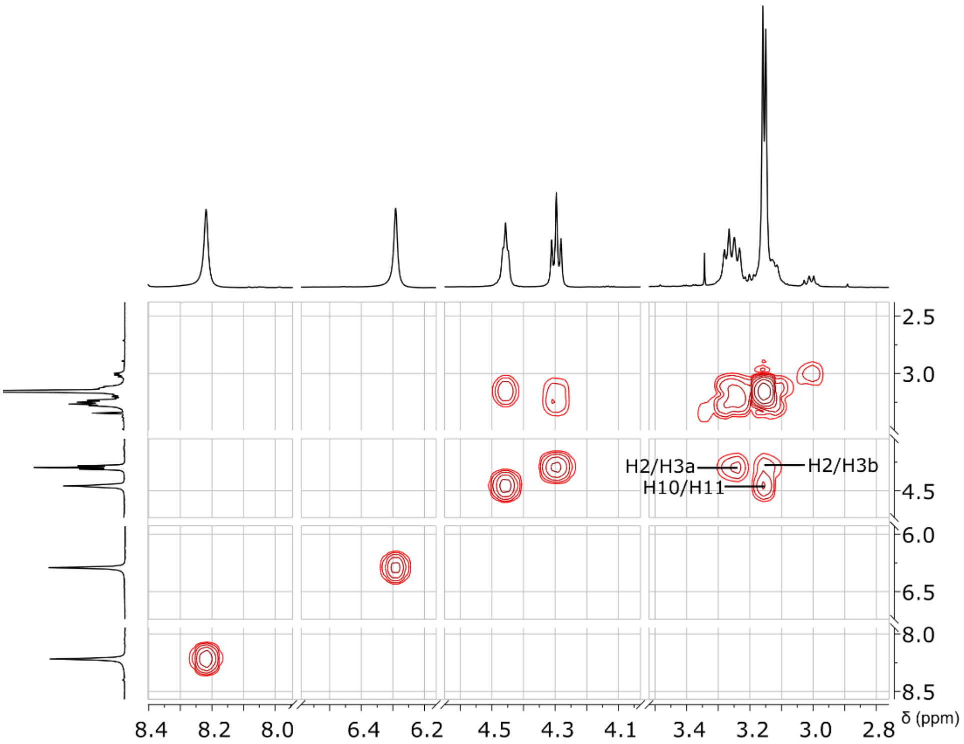

Figure S27. $^1H,^1H$-COSY spectrum of Cys-Bx, in D_2O at 25 °C.

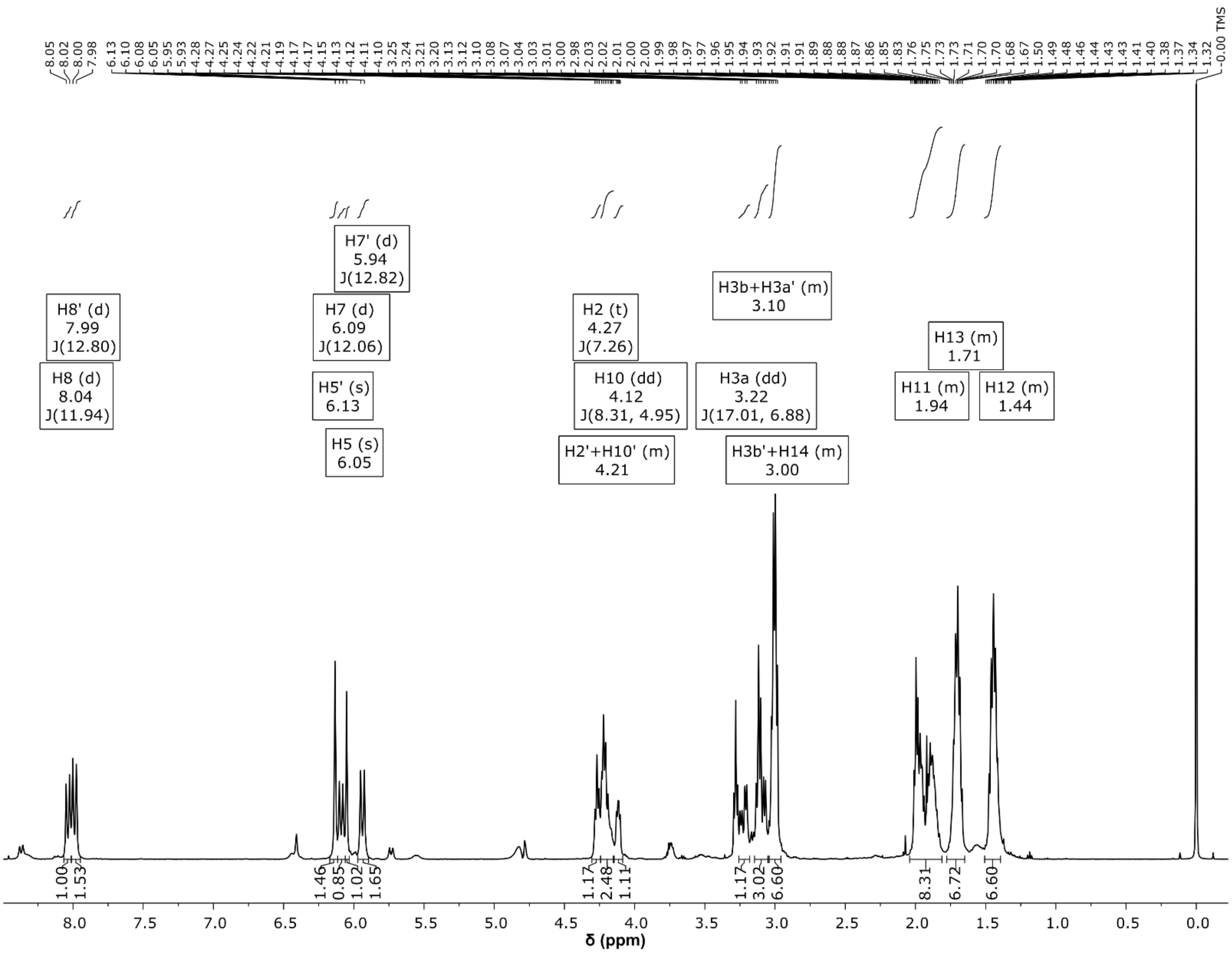

Figure S28. ^{1}H NMR spectrum of Lys-Bx, in D_2O at 25 °C.

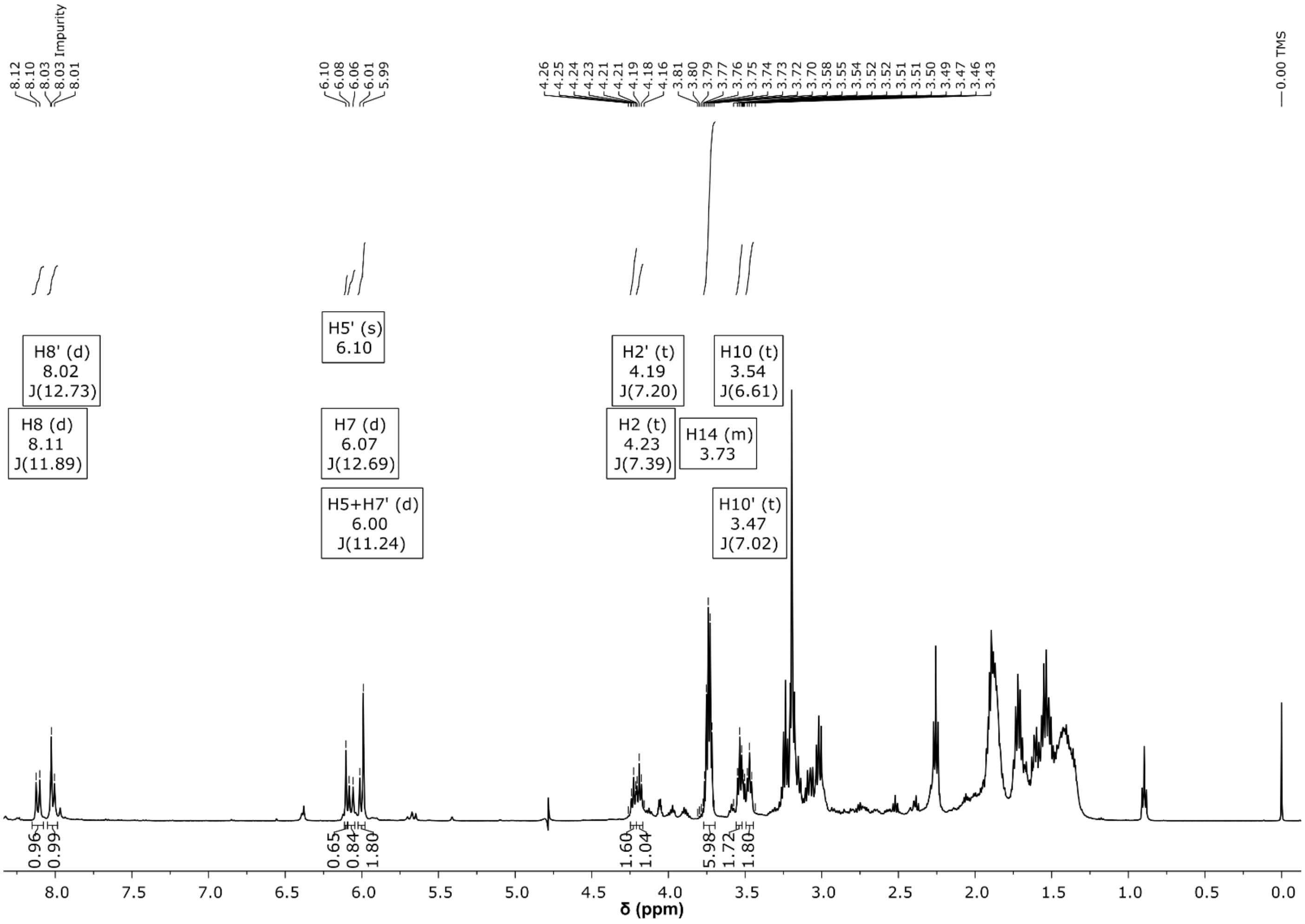

Figure S29. ^{1}H NMR spectrum of ε-Lys-Bx, in D_2O at 25 °C. It was not possible to unequivocally attribute the signals corresponding to H-3a/b and H11 – H13.

4. Ferric-reducing antioxidant power (FRAP)

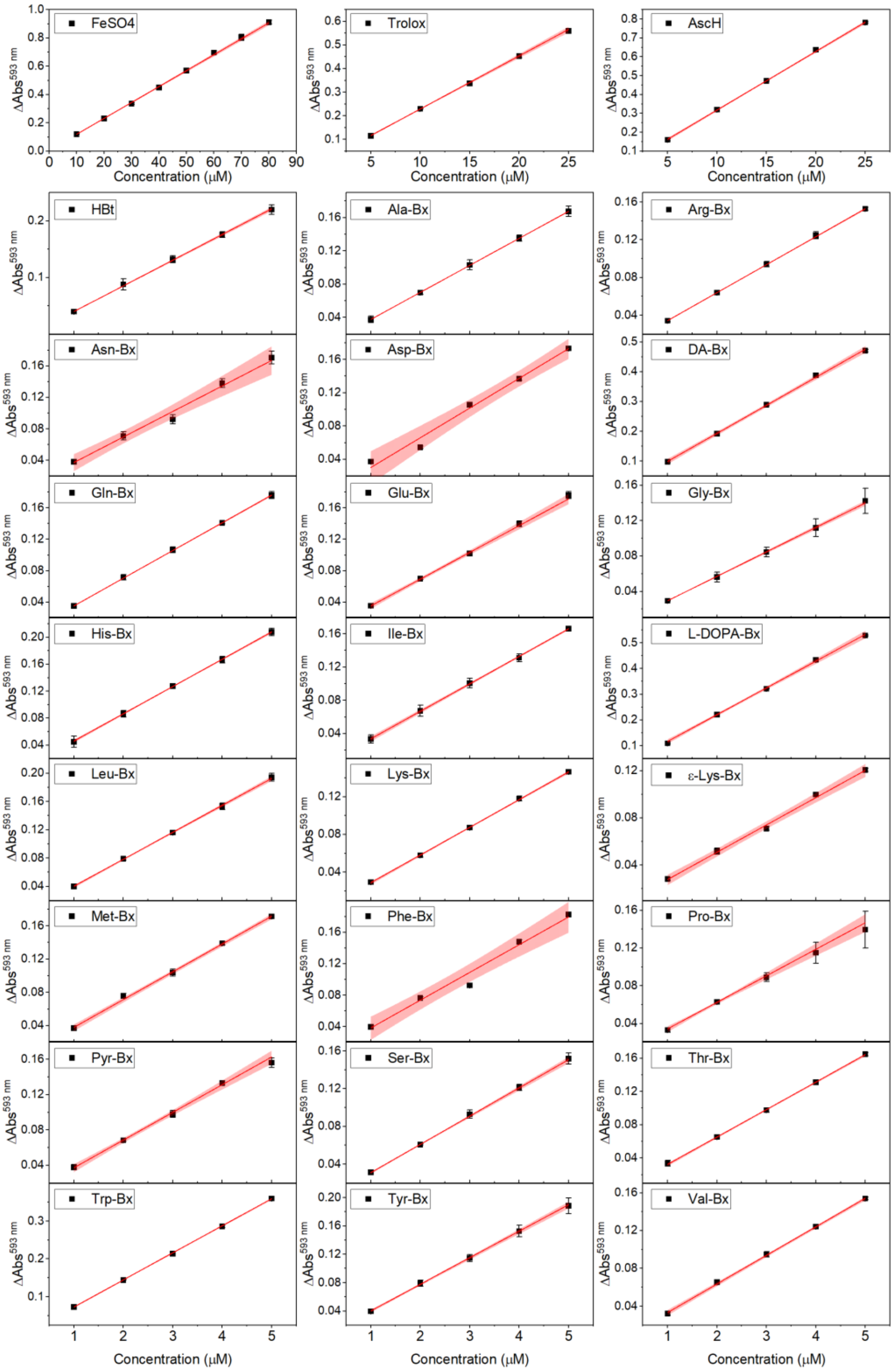

Figure S30. Change in absorbance (ΔAbs) at 593 nm due to the reduction of the (Fe(III)-TPTZ) complex to its ferrous form (Fe(II)-TPTZ) after 30 min of reaction as a function of antioxidant concentration for Trolox, ascorbic acid (AscH), betalamic acid (HBt) and betaxanthins in acetate buffer (pH 3.6, 300 mmol L^{-1}). $FeSO_4$ was used as a positive control.

5. Antiradical capacity, TEAC/ABTS•+ assay

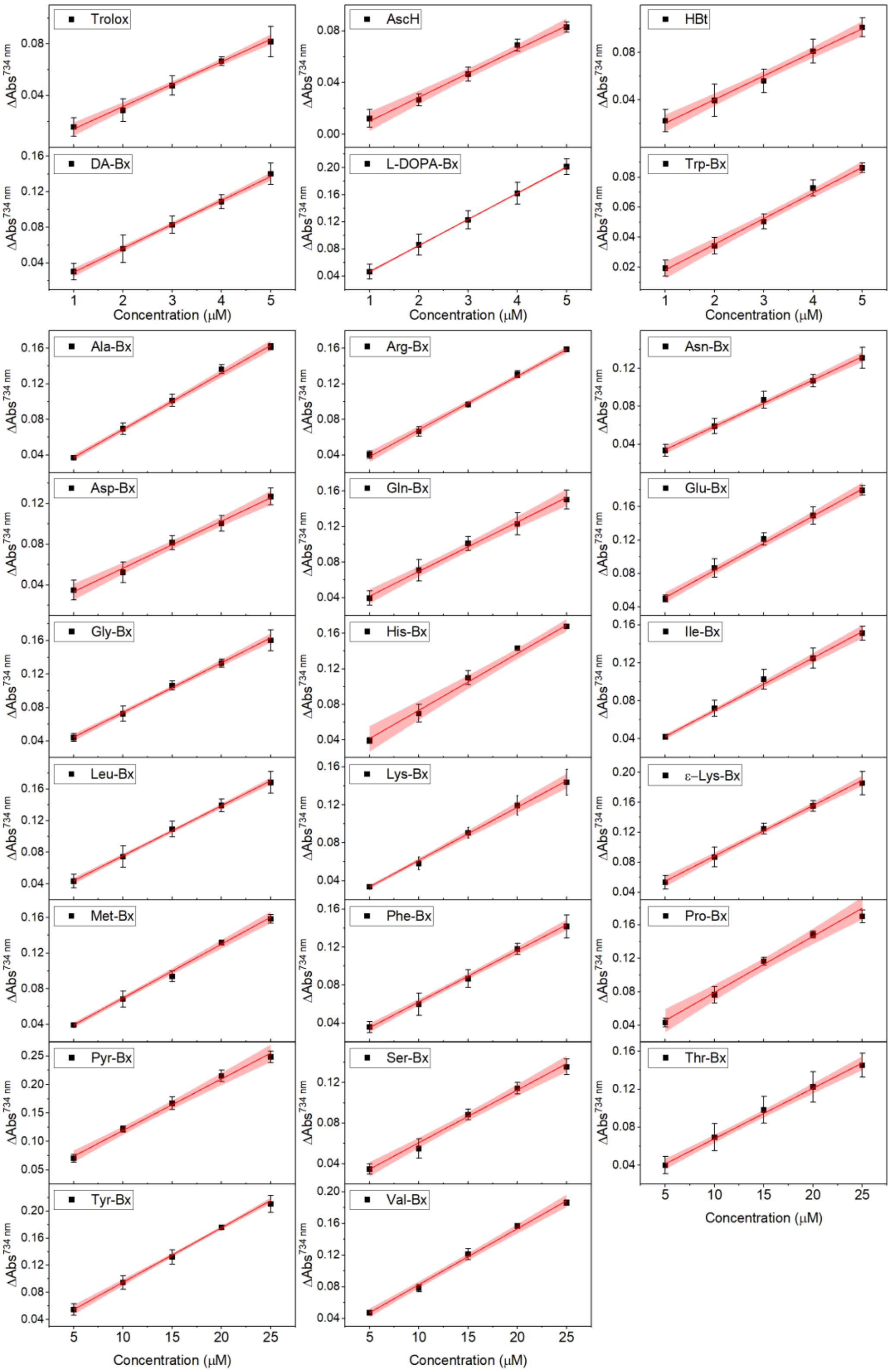

Figure S31. Change in absorbance (ΔAbs) of ABTS•+ at 734 nm after 6 min of reaction as a function of antioxidant concentration for Trolox, ascorbic acid (AscH), betalamic acid (HBt) and betaxanthins in acetate buffer (pH 3.6, 300 mmol L^{-1}).

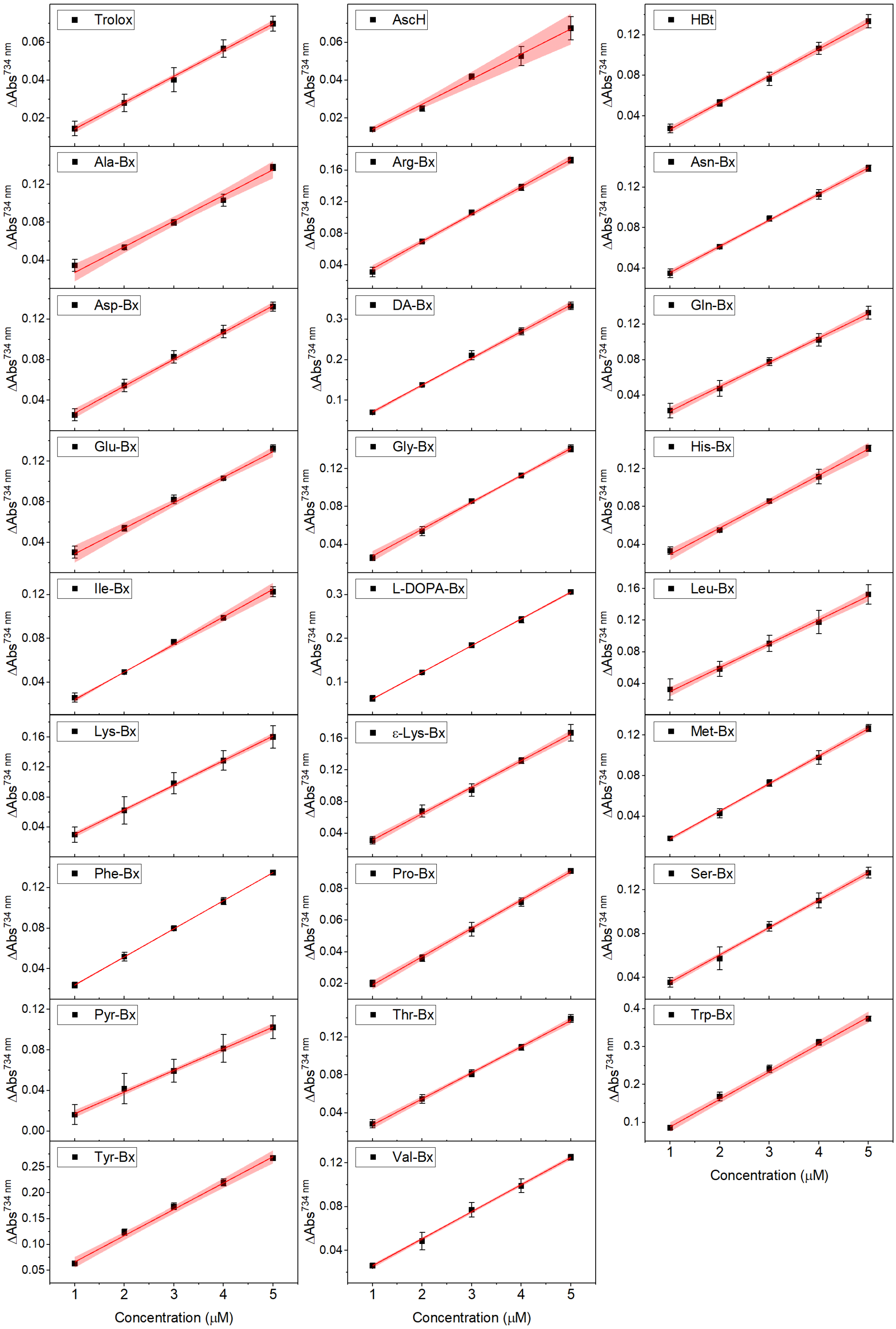

Figure S32. Change in absorbance (ΔAbs) of ABTS$^{\bullet +}$ at 734 nm after 6 min of reaction as a function of antioxidant concentration for Trolox, ascorbic acid (AscH), betalamic acid (HBt) and betaxanthins in phosphate buffer (pH 7.0, 100 mmol L^{-1}).

6. Antioxidant capacity of Cys-Bx

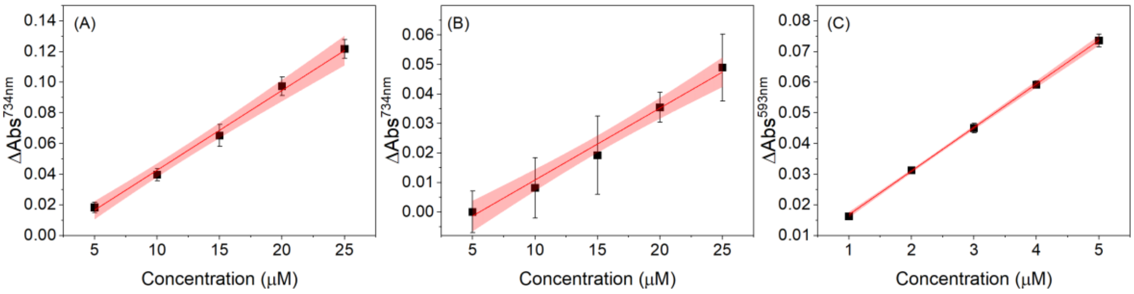

Figure S33. Change in absorbance (ΔAbs) of **(A)** ABTS$^{•+}$ at 734 nm after 6 min of reaction as a function of Cys-Bx concentration in phosphate buffer (pH 7.0, 100 mmol L^{-1}); **(B)** ABTS$^{•+}$ at 734 nm after 6 min of reaction as a function of Cys-Bx concentration in acetate buffer (pH 3.6, 300 mmol L^{-1}); **(C)** at 593 nm due to the reduction of the (Fe(III)-TPTZ) complex to its ferrous form (Fe(II)-TPTZ) after 30 min of reaction as a function of Cys-Bx concentration in acetate buffer (pH 3.6, 300 mmol L^{-1}).

7. Cyclic voltammograms

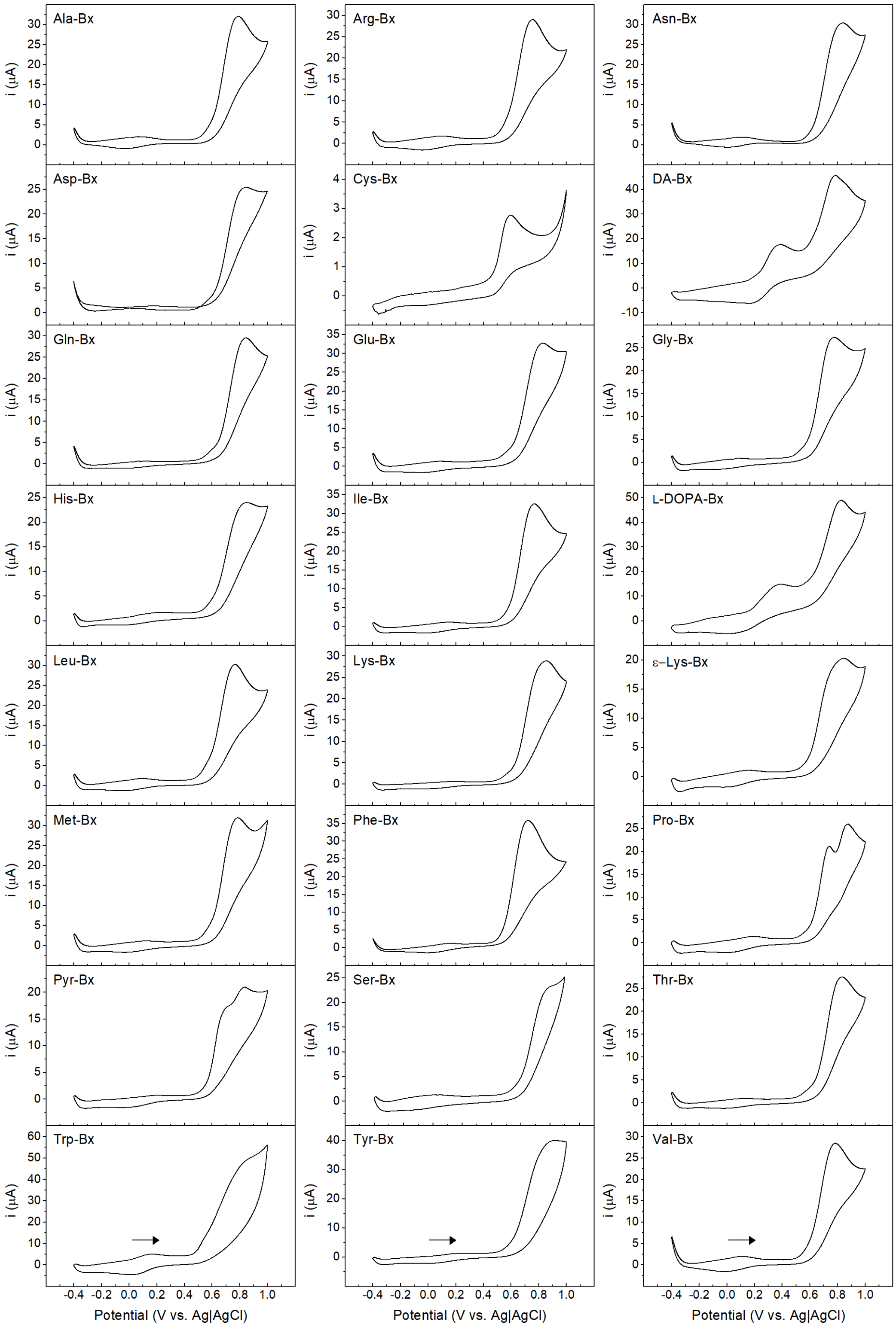

Figure S34. Cyclic voltammograms of betaxanthins obtained using screen printed vitreous carbon electrodes against an Ag/AgCl reference electrode and a carbon auxiliary electrode; potential range: –0.4 to 1.0 V, scan rate: 50 mV s^{-1}, betaxanthin concentration: 1 mmol L^{-1} in aqueous KCl (0.1 mol L^{-1}) at pH 7.0. The cyclic voltammogram of the supporting electrolyte (KCl 0.1 mol L^{-1}; background current) was subtracted from the cyclic voltammograms of the betaxanthins. The arrow indicates the beginning of the scan and the sweep direction.

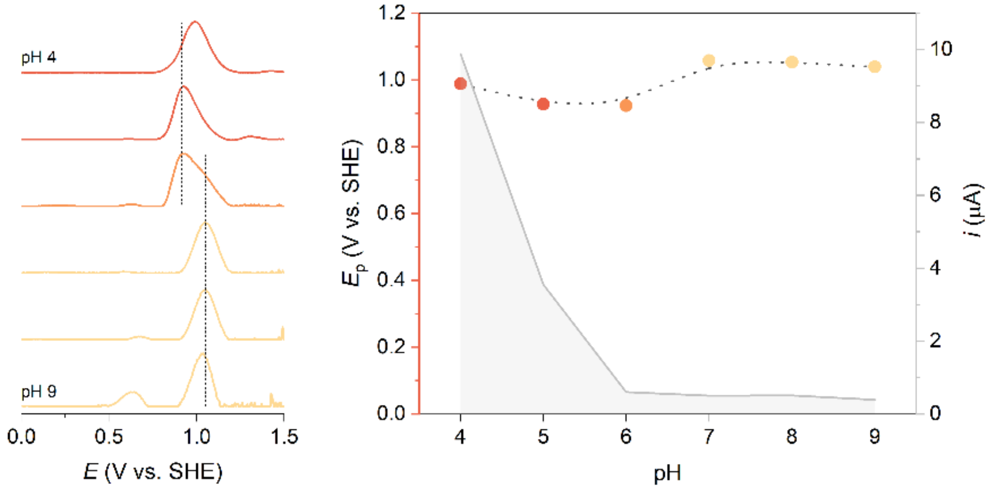

Figure S35. Background-corrected square wave voltammetry of Pro-Bx. The experiments were performed using a boron-doped diamond (BDD) working electrode, a 0.72 cm^2 Pt foil as auxiliary electrode, and an Ag/AgCl (3.0 mol L^{-1} KCl) reference electrode. All values for peak potentials (E_P) and peak current (i_P) were determined from the treated voltammograms after deconvolution of the non-linear curve fit into split-Gaussian curves.

Table S1. Values of anodic ($E_{p,a}^{I}$, $E_a^{II'}$ and $E_{p,a}^{II''}$) and cathodic ($E_{p,c}$) peak potentials, half-wave potentials ($E_{1/2}$), half-peak potentials ($E_{p/2}$) and inflection-point potentials (E_i) for betaxanthins.

	Peak potentials						
	$E_{p,a}^{I}$	$E_{p,a}^{II'}$	$E_{p,a}^{II''}$	$E_{p,c}$	$E_{1/2}^{a}$	$E_{p/2}^{b}$	E_i^{b}
**Pyrrolidine-Bx**		1.00^{c}	1.12			0.90^{d}	0.91<br>1.07^{e}
**L-DOPA-Bx**	0.68	1.11		0.29	0.49	0.96	1.02
**Dopamine-Bx**	0.68	1.07		0.45	0.56	0.93	0.99
**Trp-Bx**	0.46	1.13^{c}		0.30	0.38	0.93^{d}	0.94
**Tyr-Bx**	0.52	1.20		0.26	0.39	1.00	1.00
**Met-Bx**		1.07				0.95	0.97
**Cys-Bx**		0.89				0.80	0.81
**Phe-Bx**		1.01				0.90	0.91
**His-Bx**		1.14				0.98	0.99
**Lys-Bx**		1.14				0.99	1.00
**ε-Lys-Bx**		1.13				0.95	0.96
**Asn-Bx**		1.13				0.99	1.00
**Gln-Bx**		1.13				1.00	1.02
**Ile-Bx**		1.06				0.94	0.96
**Arg-Bx**		1.04				0.92	0.94
**Thr-Bx**		1.12				1.00	1.01
**Leu-Bx**		1.06				0.94	0.95
**Glu-Bx**		1.12				0.98	1.00
**Pro-Bx**		1.03^{c}	1.16			0.95	0.97<br>1.11^{e}
**Ser-Bx**		1.10				0.95	0.97
**Val-Bx**		1.07				0.95	0.97
**Asp-Bx**		1.13				0.99	1.00
**Ala-Bx**		1.08				0.96	0.97
**Gly-Bx**		1.06				0.94	0.96

All values are given in V. ^{a} values obtained from $E_{p,a}^{I}$ and $E_{p,c}$, where $E_{1/2} = (E_{p,a} + E_{p,c})/2$; ^{b}: values obtained from the anodic peak $E_a^{II'}$; ^{c} values of $E_a^{II'}$ are approximated because the anodic peak is unresolved; ^{d} approximated values of $E_{p/2}$; ^{e} values of E_i obtained from the second anodic peak, $E_a^{II''}$.

Table S2. Predicted pK_a values for amino acid betaxanthins.

Betaxanthin	pK_{a1} C2-COOH	pK_{a2} C6-COOH	pK_{a3} C10-COOH	pK_{a4} N9H	pK_{a5} side
Pyrrolidine-Bx	4.13	5.81	—	—	—
L-DOPA	3.81	2.35	1.40	8.22	9.33; 12.74
Dopamine	3.97	2.41	—	11.72	9.30; 13.04
Trp-Bx	3.75	2.41	1.70	8.69	—
Tyr-Bx	3.82	2.38	1.52	8.39	9.54
Met-Bx	3.76	2.38	1.60	8.71	—
Cys-Bx	3.67	2.23	1.36	6.53	—
Phe-Bx	3.78	2.39	1.64	8.62	—
His-Bx	3.72	2.28	1.41	8.81	6.46; 13.10
Lys-Bx	3.76	2.31	1.46	8.81	10.22
ε-Lys-Bx	3.94	2.65	2.06	11.95	9.29
Asn-Bx	3.81	2.36	1.46	8.28	—
Gln-Bx	3.77	2.35	1.52	8.59	—
Ile-Bx	3.68	2.35	1.62	8.74	—
Arg-Bx	1.32	3.10	1.89	8.60	12.16
Thr-Bx	3.63	2.23	1.44	7.74	—
Leu-Bx	3.76	2.40	1.65	8.82	—
Glu-Bx	3.76	1.86	2.58	8.58	4.88
Pro-Bx	4.03	5.62	3.05	—	—
Ser-Bx	3.71	2.27	1.40	7.76	—
Val-Bx	3.68	2.32	1.58	8.70	—
Asp-Bx	3.78	2.34	1.38	8.53	5.07
Ala-Bx	3.86	2.41	1.52	8.75	—
Gly-Bx	3.78	2.33	1.45	9.04	—
